# Molecular characteristics and prognostic significances of lysosomal-dependent cell death in kidney renal clear cell carcinoma

**DOI:** 10.18632/aging.205639

**Published:** 2024-03-07

**Authors:** Shunliang He, Jiaao Sun, Hewen Guan, Ji Su, Xu Chen, Zhijun Hong, Jianbo Wang

**Affiliations:** 1 Department of Urology, First Affiliated Hospital of Dalian Medical University, Dalian, Liaoning, China; 2 Department of Dermatology, First Affiliated Hospital of Dalian Medical University, Dalian, Liaoning, China; 3 Department of Urology, Central Hospital of Benxi, Benxi, Liaoning, China; 4 Department of General Surgery, First Affiliated Hospital of Dalian Medical University, Dalian, Liaoning, China; 5 The Health Management Center, First Affiliated Hospital of Dalian Medical University, Dalian, Liaoning, China

**Keywords:** kidney renal clear cell carcinoma, lysosomal-dependent cell death, molecular classification, prognosis significance, therapeutic opportunities

## Abstract

Lysosomal-dependent cell death (LDCD) has an excellent therapeutic effect on apoptosis-resistant and drug-resistant tumors; however, the important role of LDCD-related genes (LDCD-RGs) in kidney renal clear cell carcinoma (KIRC) has not been reported. Initially, single-cell atlas of LDCD signal in KIRC was comprehensively depicted. We also emphasized the molecular characteristics of LDCD-RGs in various human neoplasms. Predicated upon the expressive quotients of LDCD-RGs, we stratified KIRC patients into tripartite cohorts denoted as C1, C2, and C3. Those allocated to the ambit of C1 evinced the most sanguine prognosis within the KIRC cohort, underscored by the acme of LDCD-RGs scores. This further confirms the significant role that LDCD-RGs play in both the pathophysiological foundation and clinical implications of KIRC. In culmination, by virtue of employing the LASSO-Cox analytical modality, we have ushered in an innovative and avant-garde prognostic framework tailored for KIRC, predicated on the bedrock of LDCD-RGs. The assemblage of KIRC instances was arbitrarily apportioned into constituents inclusive of a didactic cohort, an internally wielded validation cadre, and an externally administered validation cohort. Concurrently, patients were dichotomized into strata connoting elevated jeopardy synonymous with adverse prognostic trajectories, and conversely, diminished risk tantamount to favorable prognoses, contingent on the calibrated expressions of LDCD-RGs. Succinctly, our investigative findings serve to underscore the cardinal capacity harbored by LDCD-RGs within the KIRC milieu, concurrently birthing a pioneering prognostic schema intrinsically linked to the trajectory of KIRC and its attendant prognoses.

## INTRODUCTION

Renal cell carcinoma (RCC), one of the leading causes of cancer-related deaths worldwide, has long been recognized as a major threat. Among renal malignancies, kidney renal clear cell carcinoma (KIRC) accounts for 80% and remains the most prevalent type of kidney cancer [1–3]. KIRC is a highly vascularized pathological subtype of RCC, originating from the proximal tubular epithelial cells of the kidney unit [2, 3]. However, its exact etiology remains unclear, possibly involving factors such as smoking, obesity, occupational exposure to carcinogens, and genetic factors like tumor suppressor gene loss [4]. In the United States, RCC ranks eighth among malignant diseases [5], with approximately 70% of kidney cancers being diagnosed at a localized or locally advanced stage, allowing for surgical resection. Nevertheless, about one-third of patients still experience distant metastasis [6]. Unfortunately, metastatic RCC has shown high resistance to traditional radiation and chemotherapy, making molecular targeted therapy and immunotherapy the mainstays of advanced kidney cancer treatment over the past decade. Nevertheless, the lack of comprehensive understanding of KIRC’s underlying mechanisms and the emergence of resistance to targeted drugs pose ongoing challenges [2, 3, 7, 8]. Therefore, it is crucial to develop new prognostic features to more accurately predict KIRC prognosis and identify additional suitable therapeutic targets to enrich the arsenal of targeted drugs.

As we all know, regulatory cell death (RCD) plays an important role in tumorigenesis and development. Cell death is a natural biological process and there are two main types: necrosis and apoptosis. In tumors, regulatory apoptosis is essential for maintaining normal tissue structure and preventing tumor development, such as apoptosis, pyrodeath, necrotic apoptosis, iron death, entosis, NETosis, parthanatos and lysosomal dependent cell death and so on [9, 10]. Lysosomal-dependent cell death (LDCD) represents a cellular demise process in which hydrolases (specifically cathepsins) or iron, released due to lysosomal membrane permeabilization, play a pivotal role. This mode of cell death is primarily distinguished by the rupture of lysosomes. When cells encounter lysosomal detergents, dipeptide methyl esters, lipid metabolites, and reactive oxygen species (ROS), it leads to the disruption of lysosomal integrity, causing a significant release of hydrolases. This event ultimately culminates in LDCD. Cathepsin is a key player in LDCD, and inhibiting its expression or activity can effectively diminish the incidence of LDCD. The permeabilization of lysosomal membranes can additionally trigger substantial cellular death signaling pathways in instances of apoptosis, autophagy-dependent cell death, and Ferroptosis, thereby adding intricacy to the landscape of cell death pathways [9]. Lysosomes are physiologically involved in cellular homeostasis, dysregulation of which has been implicated in various human diseases, including cancer [11]. Changes in lysosome function can alter the occurrence and development of tumors and the clinical outcome of patients [12]. Considering the crucial role of lysosomal function in cancer cells, scientists have created an array of small molecule compounds that specifically target lysosomes, which possess the ability to either provoke lysosomal membrane permeability or disrupt lysosomal function, thereby effectively eradicating tumor cells [13–15]. Given the significant involvement of LDCD in oncogenesis, a comprehensive investigation into its implications within the context of KIRC is warranted. As of present, a discernible void exists within the literature pertaining to the intricate interplay between LDCD and KIRC. The comprehensive characterization of LDCD-related genes (LDCD-RGs) determinants in the milieu of KIRC pathogenesis stands as an aspect yet to attain comprehensive clarification. Moreover, the potential of these determinants to prognosticate clinical outcomes and dictate optimal chemotherapeutic strategies for individuals afflicted with KIRC remains enigmatic. Therefore, according to the expression characteristics of LDCD-RGs to stratify management of KIRC patients is of great value and significance.

In this study, we performed an in-depth analysis of the expression levels and genomic variants of LDCD-RGs in human tumors. We successfully classified the KIRC patients into 3 distinct subgroups based on the scores of the LDCD-RGs. This categorization helps us to understand the differences in prognosis, immune microenvironment, and response to therapeutic drugs among the 3 subgroups of KRIC patients. In addition, based on the LDCD-RGs, we developed a novel prognostic model for KIRC patients. In conclusion, this study provided novel insights into the role of LDCD-RGs in KIRC. By elaborating the key role of LDCD-RGs in KIRC, it can help clinical medical workers to better understand the complex pathophysiological changes and processes of KIRC.

## METHODS

### Data collection and processing

A total of 255 LDCD-RGs were downloaded from the Genecard website (https://www.genecards.org/) using the search term “lysosomal-dependent cell death” (Supplementary Table 1). Transcriptional and clinical prognostic data for TCGA-KIRC were downloaded and organized from the TCGA official website, and additional samples were added from the ArrayExpress database to augment the KIRC dataset. After Log transformation and SVA batch processing [16, 17], a total of 640 KIRC transcriptional profiles (TCGA-KIRC cohort + E-MTAB-1980 cohort) were obtained, of which 627 had complete follow-up information for prognosis-related analysis in this study. Pan-cancer genomic and transcriptomic data were also downloaded from the TCGA database for comprehensive characterization of LDCD genes across various cancers, and corresponding follow-up information was collected for pan-cancer prognostic analysis, following a methodology similar to previous reports. Additionally, single-cell RNA sequencing (scRNA-seq) data was acquired from GSE156632 dataset [18], which involved seven KIRC samples and five adjacent normal samples.

### scRNA-seq analysis

The analysis of single-cell data was primarily conducted using the Seurat package. The CreateSeuratObject function was employed to read the GSE156632 dataset, with the following parameters set: min.cells = 3, min.features = 200, nCount_RNA ≥1000, nFeature_RNA ≥200, nFeature_RNA ≤6000, percent.mt ≤20. Classic single-cell data analysis procedures and multiple algorithmic gene set scoring predictions were utilized to investigate LDCD features at the single-cell level. The following functions were employed for single-cell data cleaning: NormalizeData, FindVariableFeatures, ScaleData, RunPCA, RunHarmony, JackStraw, ScoreJackStraw, FindNeighbors, FindAllMarkers, RunTSNE. Cell annotations were based on the following criteria: Epithelial cell markers included KRT8, KRT18, EPCAM; Myeloid cell markers included CD14, CD68; Fibroblast cell markers included TAGLN, RGS5, MYL9, ACTA2; T/NK cell markers included CD2, CD3D, CD3E, CD3G, NKG7, GNLY; Endothelial cell markers included ENG, PECAM1, VWF, CDH5; B cell markers included MS4A1, CD79A, CD79B. Five single-cell gene set scoring prediction algorithms were utilized: Add, AUCell, UCell, singscore, and GSVA. Additionally, a novel custom single-cell gene set scoring prediction algorithm was developed, which involved scaling and 0-1 normalization of the results from the aforementioned five algorithms (scoring = (*x* − min(*x*))/(max(*x*) − min(*x*))), followed by summation. This approach effectively eliminated the impact of non-biological factors. Visualization of single-cell data was primarily achieved through the ggviolin and FeaturePlot functions.

### Identification of prognosis-related LDCD genes in the KIRC cohort

Firstly, the expression profiles of 255 LDCD genes were extracted from 627 KIRC samples with complete prognostic information. These expression profiles were integrated with patients’ survival time and status data, and prognosis-related LDCD genes were identified using univariate Cox regression analysis with *P*-values adjusted using the Benjamini-Hochberg method (*P* < 0.05).

### Molecular subtyping of KIRC based on prognosis-related LDCD genes

To define the expression patterns of LDCD genes in KIRC, a method similar to previous research was used by comparing gene expression characteristics between tumor tissues and adjacent normal tissues [19]. As the E-MTAB-1980 cohort lacked adjacent normal tissues, the analysis for molecular subtyping was performed using only the TCGA-KIRC cohort. Unsupervised clustering using the “hclust” function and GSVA scoring using the “gsva” function were employed to identify molecular subtypes. The differences in LDCD scores among different molecular subtypes were assessed using the Kruskal-Wallis test. Survival curves comparing the prognostic differences among molecular subtypes were plotted using the survival and survminer packages. The ggplot2 and ggpubr software packages were used to analyze whether the LDCD-RGs scores were statistically different among the 3 subgroups.

### Exploration of intrinsic molecular characteristics and therapeutic sensitivity of different LDCD molecular subtypes

Classic signaling pathways related to metabolism, immunity, and cell death were collected and compiled from MsigDb and previous literature reports [20]. GSVA was used to calculate pathway scores for each tumor sample [21], and the Kruskal-Wallis test was employed to compare clinical outcomes among different LDCD molecular subtypes. Furthermore, the immune microenvironment of different LDCD molecular subtypes was explored through analysis of immune cell infiltration and immune checkpoint expression levels. The TIMER2.0 platform [22] was utilized to predict immune cell infiltration abundance in the TCGA-KIRC cohort. Heatmaps were generated using the pheatmap and limma packages to visualize immune profiles associated with LDCD molecular subtypes, and statistical differences were evaluated using the Kruskal-Wallis test. The expression levels of recognized immune checkpoints [23] were also compared among different LDCD molecular subtypes.

### Investigation of the correlation between LDCD-RGs and immune cell infiltration and immune function in KIRC

GSVA was employed to predict the immune cell infiltration and immune-related functions for each KIRC patient. Spearman’s correlation was used to analyze the association between LDCD gene expression and immune cell infiltration or immune function. Correlation heatmaps were plotted using the ggplot2 package, with “^*^” indicating *P* < 0.05 and “^**^” indicating *P* < 0.01. Bar plots and scatter plots were created using the ggplot2 and ggstatsplot packages to visualize the correlation between LDCD gene set scores and immune cell infiltration or immune function.

### Development and validation of an LDCD-related tumor prognostic model

An LDCD-related tumor prognostic model was developed based on prognosis-related LDCD genes. The TCGA-KIRC cohort was randomly divided into training and internal validation sets at an approximate ratio of 1:1 (264 tumor samples in the training set and 262 tumor samples in the internal validation set 1). All TCGA-KIRC samples (526 tumor samples) were used as internal validation set 2, and the E-MATB-1980 cohort (101 tumor samples) served as the external validation set. The training set was used for LASSO-multivariate Cox regression to construct the prognostic model. Each tumor patient was assigned a riskscore value, predicted using the “predict” function. Based on the median riskscore value in the training set, patients were categorized into high-risk and low-risk groups. Similar approaches were applied to internal validation set 1, internal validation set 2, and external validation set. To assess the clinical significance of the model, survival curves were plotted using the survival and survminer packages. ROC curves were generated, and corresponding AUC values were calculated using the survival ROC package to determine the prognostic discrimination of the survival curves. Immune cell infiltration in each sample was evaluated based on the TIMER2.0 platform, and differences in immune cell infiltration between high-risk and low-risk groups were compared. Finally, we utilized the random forest algorithm to calculate the prognostic contribution importance of each model gene for KIRC disease. We combined this analysis with information from the BEST website (https://rookieutopia.com/app_direct/BEST/) to explore the clinical relevance of the genes, emphasizing their central role. HPA website was utilized to explore the protein level of hub gene in KIRC.

### Availability of data and materials

The datasets analyzed in this work may be found in the Supplementary Materials or contact with the corresponding author.

## RESULTS

### scRNA analysis

**Figure 1:**
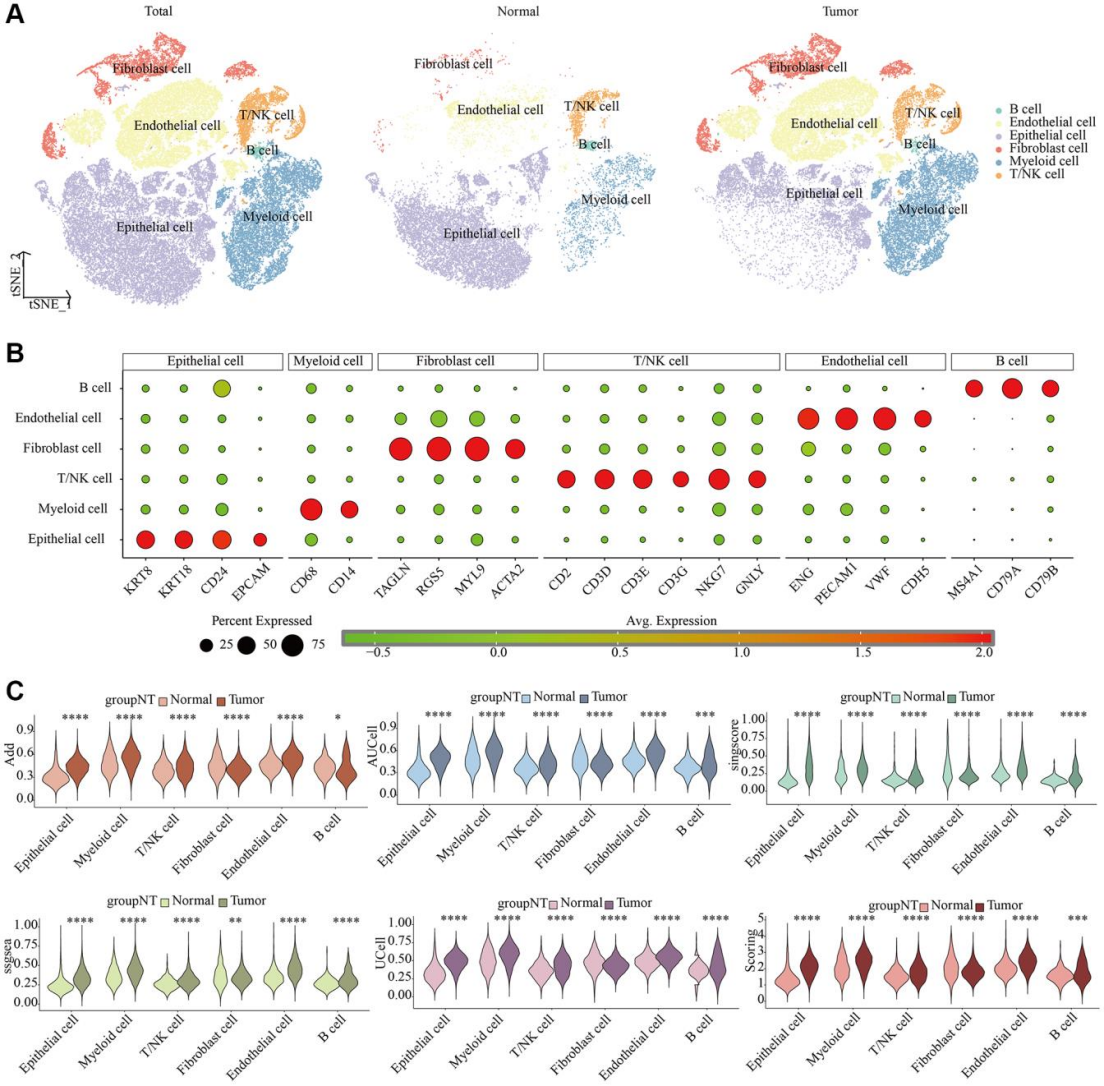
**Single-cell characteristics of LDCD signal in KIRC and para-cancer samples.** (**A**) t-SNE dimensionality reduction plot. (**B**) Cell annotation. (**C**) Prediction of LDCD signal based on six algorithms. Abbreviations: LDCD: Lysosomal-dependent cell death; KIRC: kidney renal clear cell carcinoma.

We depicted single-cell dimensionality reduction maps for both KIRC and adjacent normal samples, identifying epithelial cells, endothelial cells, fibroblasts, and some immune cell types (Figure 1A). These cell types were annotated based on classical cell markers, as detailed in Figure 1B. To comprehensively reveal the LDCD signal features of different cell types in KIRC, we employed six evaluation methods for quantitative analysis. As illustrated in Figure 1C and Supplementary Figure 1, the results consistently indicated that cells in the tumor tissue exhibited a relatively high level of LDCD signal intensity, regardless of the algorithm used.

### Pan-cancer characteristics of LDCD-RGs

**Figure 2:**
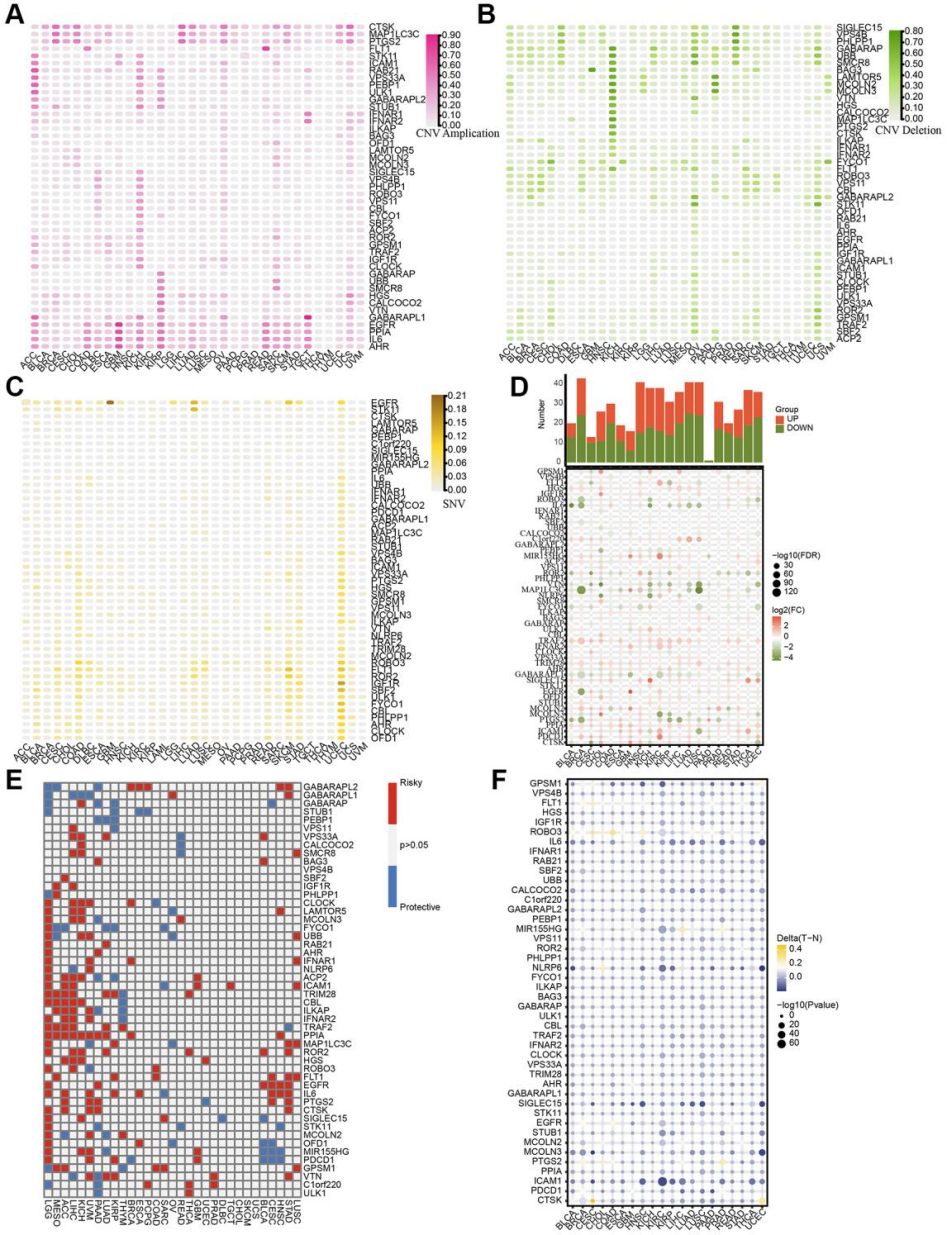
**Pan-cancer characteristics of 50 LDCD-RGs.** (**A**, **B**) Depiction of CNV mutations (gain and loss frequencies) within the cohort of LDCD-RGs manifesting in human tumors. (**C**) Heatmap was utilized to depict the SNV data of the LDCD-RGs in human tumors. (**D**) Depiction of mRNA expression magnitudes for LDCD-RGs. (**E**) Survival landscape assessment was performed on the set of LDCD-RG in human cancers. Genes displaying a *P*-value exceeding 0.05 are depicted in white, blue represents protective, red represents risk. (**F**) The methylation landscape governing LDCD-RGs in human tumors. Abbreviations: LDCD-RGs: LDCD-related genes; CNV: Copy Number Variation.

We initially identified a set of 50 LDCD-RGs linked to the prognosis of KIRC through the application of univariate Cox regression analysis (Supplementary Table 2). In order to comprehensively depict the pan-cancer characteristics of above 50 LDCD-RGs, we conducted genomic and transcriptomic-level analyses using the TCGA pan-cancer cohort. A meticulous inquiry encompassing copy number variation (CNV), stable nuclear variants (SNV), mRNA expression quantification, methylation patterns, along with their nascent functional implications for LDCD-RGs was undertaken. Depiction of CNV mutations within the cohort of LDCD-RGs manifest in neoplastic entities was documented (Figure 2A, 2B), thereby illustrating variances in CNV frequencies across distinct malignancy subtypes. Remarkably heightened frequencies of gain-of-function CNV mutations were discerned within the ambit of ACC, KICH, and KIRP for a majority of LDCD-RGs. It merited emphasis that conspicuous amplification-centric CNV mutation frequencies were conspicuously noted for key genes including EGFR, PPIA, IL6, and AHR, spanning various malignancy categories. Additionally, outcomes pertaining to CNV-associated loss frequencies unveiled significantly augmented rates of loss-of-function mutations amongst LDCD-RGs in the purview of KICH, OV, UCS, PCPG, and READ, with KICH assuming pronounced prominence relative to other gene counterparts. It is worth noting, however, that a predominant proportion of LDCD-RGs exhibited subdued frequencies of loss-of-function mutations across over fifty percent of the cancer classifications. Moreover, an observation of heightened SNV mutation instances within human solid tumors was ascertained particularly within the context of UCEC. Furthermore, the predilection toward low-frequency SNV mutations was a prevailing trend amidst the majority of LDCD-RGs (Figure 2C). Recognizing the inherent pertinence of aberrant gene expression to oncogenesis, we proffered a depiction of mRNA expression magnitudes for LDCD-RGs across a spectrum of human tumor types (Figure 2D). Evident by comparison to their cognate normal tissue analogs, discernible upregulation was registered across a slew of tumor tissues for PPIA, TRAF2, TRIM28, and HGS. Conversely, a pervasive decrement was witnessed in the expression of GABARAPL1, MAP1LC3C, VTN, and IL6 in nearly all tumor specimens relative to their paired normal tissue counterparts. This insight duly impelled an exploration into the nexus between gene expression levels and the temporal trajectory of patient survival in the throes of tumorigenicity, thereby substantiating the multifaceted roles of LDCD-RGs as both instigators of vulnerability and sentinels of safeguarding in disparate solid tumor contexts (Figure 2E). On a broader note, the tenor of risk was notably orchestrated by the majority of LDCD-RGs, a predilection accentuated particularly within LGG, MESO, ACC, KICH, and UVM. Contemplating the canonical role of aberrant gene methylation as a recurrent epigenetic hallmark in tumorigenesis, typified by overarching genome-wide hypomethylation tendencies within oncogenes juxtaposed against aberrant CpG island-specific hypermethylation proclivities within tumor suppressor genes, we embarked upon an inquisition into the methylation landscape governing LDCD-RGs within human neoplasms (Figure 2F). The ensuing revelations underscored the pervasiveness of hypomethylation across nearly all LDCD-RGs in the context of human cancers, evincing comprehensive hypomethylated states within the gamut of LDCD-RGs specifically within the precincts of KIRC.

### Patients with KIRC were grouped based on LDCD-RGs expression levels

**Figure 3:**
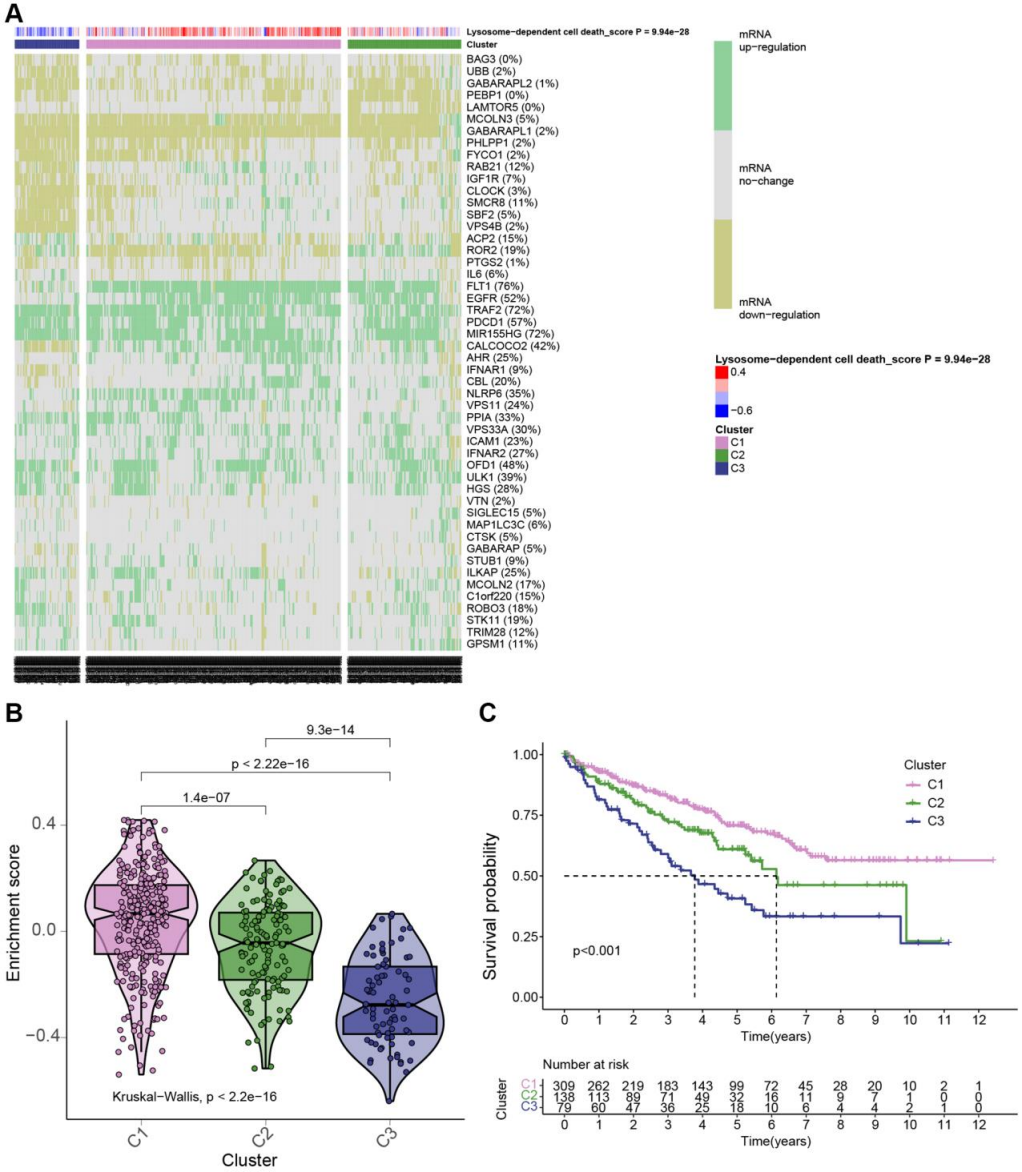
**KIRC patients were grouped in 3 clusters based on LDCD-RGs expression levels.** (**A**) Based on TCGA data, a heatmap generated through cluster analysis illustrates three subgroups of KIRC: Highly Active LDCD (C1), Normal LDCD (C2), and Suppressed LDCD (C3), categorized according to the mRNA expression levels of 50 LDCD-RGs. (**B**) Violin plots created using the “ggpubr” package depict the enrichment scores of the three subgroups in descending order: C1, C2, and C3. (**C**) Comparison of survival curves among the three KIRC subgroups reveals differing survival times (C1 > C2 > C3). The pink line represents C1; the green line represents C2; and the black line represents C3. The horizontal axis denotes time (years), while the vertical axis represents survival rate. Abbreviations: LDCD-RGs: LDCD-related genes; LDCD: Lysosomal-dependent cell death; KIRC: kidney renal clear cell carcinoma.

Then, to comprehensively investigate the pivotal role of the 50 LDCD-RGs in the initiation and progression of KIRC, we partitioned the KIRC samples sourced from TCGA into three distinct subgroups, namely cluster 1, cluster 2, and cluster 3. This partitioning was executed based on the expression levels of LDCD-RGs, as demonstrated in Figure 3A. Cluster 1 (C1) encompassed KIRC patients exhibiting active LDCD, Cluster 3 (C3) encompassed those with suppressed LDCD, and Cluster 2 (C2) consisted of KIRC patients demonstrating normal LDCD activity. Furthermore, we performed an in-depth analysis to enhance our understanding of the expression scores of LDCD-RGs across these three clusters, as illustrated in Figure 3B. Subsequent to this analysis, we proceeded to construct survival curves for each of the three cluster subgroups. Intriguingly, our findings unveiled that KIRC patients belonging to Cluster C1 showcased the highest overall survival rate, whereas those falling within Cluster C3 displayed the lowest overall survival rate (Figure 3C). Importantly, the observed disparities in survival curves among these three subgroups were underpinned by statistically significant differences. This underscores the considerable clinical implications of the LDCD-RGs score in the prognosis and post-treatment trajectory of KIRC patients, thereby establishing it as a noteworthy protective indicator for KIRC.

### Correlations between the LDCD-RGs score and the classical cancer-related metabolic, immune and cell death pathways

**Figure 4:**
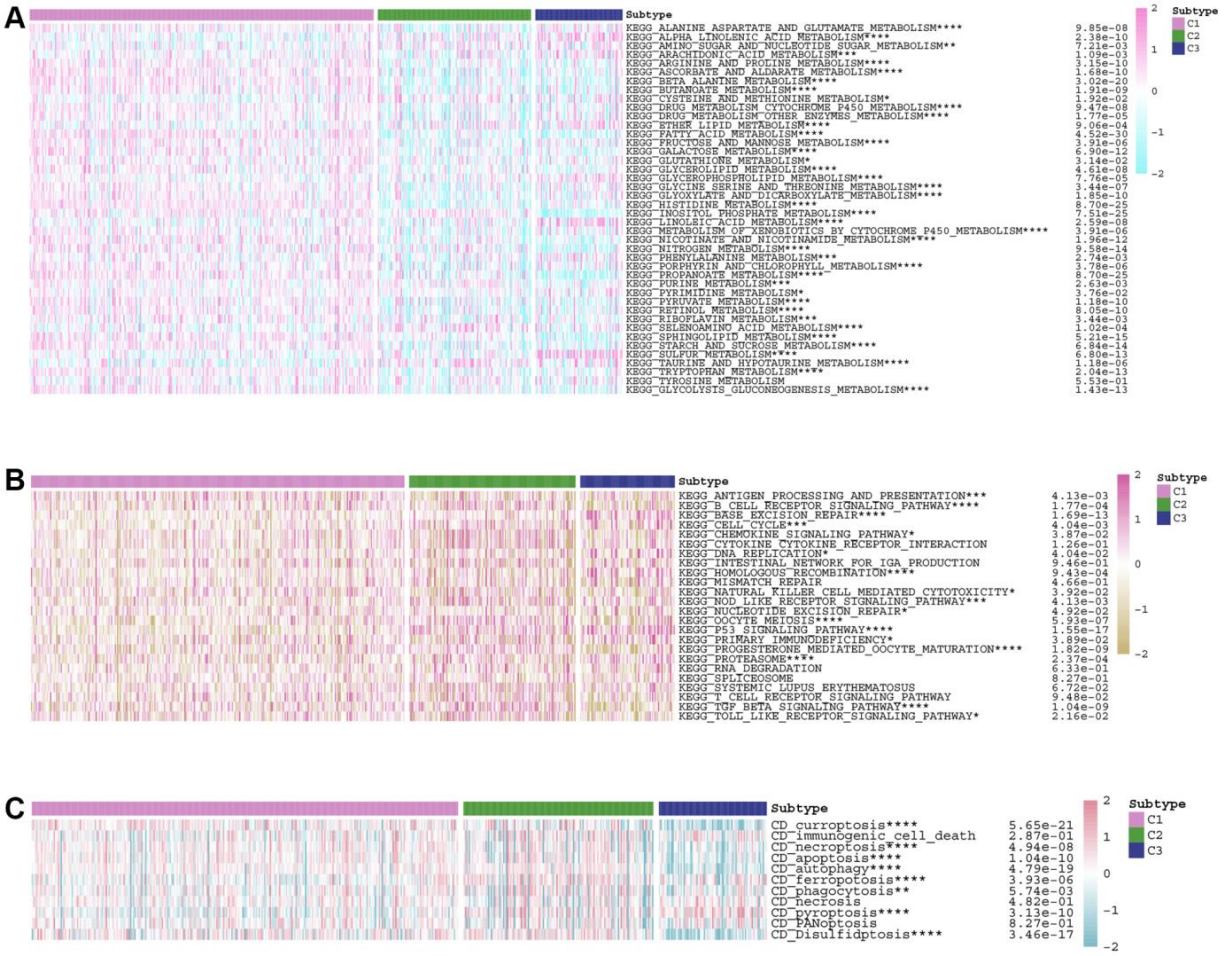
**Correlations between the LDCD-RGs score and the classical cancer-related metabolic, immune and cell death pathways.** (**A**) Analysis of differences in activity of classical metabolic pathways among 3 subgroups of KIRC. (**B**) Analysis of differences in activity of classical immune pathways among 3 subgroups of KIRC. (**C**) Analysis of differences in activity of classical cell death pathways among 3 subgroups of KIRC. (^*^p < 0.05; ^**^p < 0.01; ^***^p < 0.001; ^****^p < 0.0001). Abbreviations: LDCD-RGs: LDCD-related genes; KIRC: kidney renal clear cell carcinoma.

Given the pivotal role of LDCD-RGs in the etiology and progression of KIRC, this study delves into the mechanistic underpinnings by which LDCD-RGs exert their influence on the pathogenesis of KIRC. An extensive analysis was conducted to discern variations in classical cancer-associated metabolic channeling within the tripartite KIRC subgroups (Figure 4A). Aside from KEGG_TYROSINE_METABOLISM, a gamut of metabolically pertinent pathways exhibited statistically significant differences across the three KIRC subgroups. Evidently, the C1 subgroup displayed heightened activity in numerous cancer-linked pathways relative to the C2 and C3 counterparts. Instances include KEGG_FATTY_ACID_METABOLISM, KEGG_INOSITOL_PHOSPHATE_METABOLISM, and KEGG_GLYCOLYSIS_GLUCONEOGENESIS_MET ABOLISM. Conversely, a subset of cancer-associated metabolic pathways such as KEGG_LINOLENIC_ACID_METABOLISM, KEGG_SULFUR_METABOLISM and KEGG_ALPHA_LINOLENIC_ACID_METABOLISM evinced subdued activity within the C3 subgroup. These compelling findings propose the potential of LDCD-RGs to modulate the prognostic trajectory of KIRC in affected patients by exerting either a promotive or inhibitory effect on tumor-associated metabolic pathways. Further exploration encompassed an evaluation of the interplay between the activity of canonical immune-related pathways and LDCD-RGs in KIRC patients (Figure 4B). Multiple immune pathways, including antigen presentation response, T-cell receptor signaling pathway, and B-cell receptor signaling pathway, exhibited markedly aberrant activation patterns across the three subgroups. On the whole, LDCD-RGs participate in the orchestration of immune-related pathways in KIRC patients, thereby exerting an influence on the clinical prognosis of the condition. This intricate mechanism warrants further investigation to elucidate its specific modalities. Given the crucial function of cell death patterns in the genesis and evolution of tumors, an in-depth analysis was pursued to discern disparities in the activity of cell death pathways among the three KIRC subgroups (Figure 4C). In addition to immunogenic_cell_death, necrosis, and PANoptosis cell death pathways, residual necrosis demonstrated a substantive correlation with the LDCD-RGs score. For instance, positive correlations were established between LDCDD-RGS scores and curroptosis as well as disulfidptosis, while an inverse correlation emerged with pyroptosis, as evidenced by the LDCD-RGs scores.

### Conducting drug sensitivity analysis across the three KIRC subgroups

**Figure 5:**
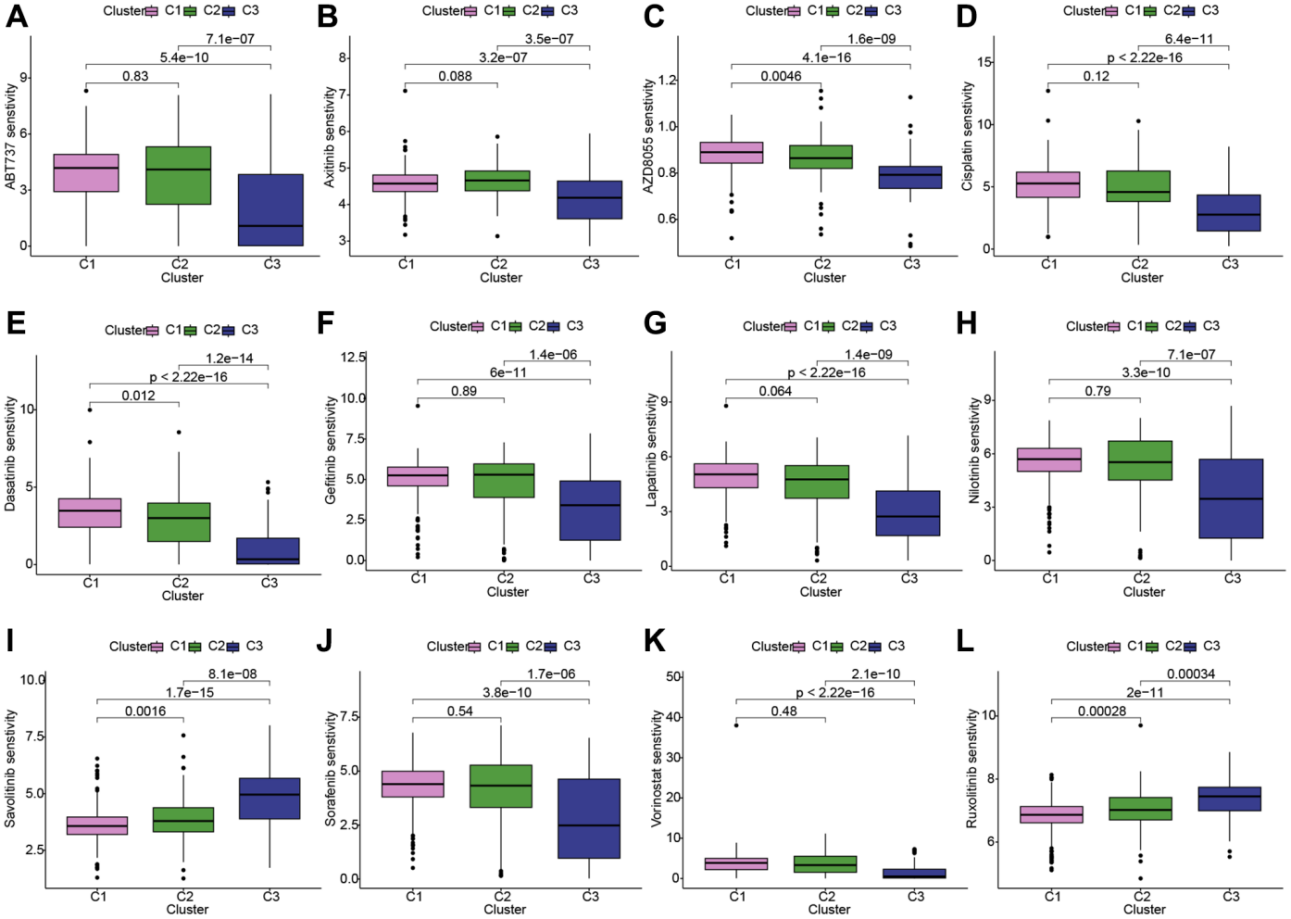
**Conducting drug sensitivity analysis across the three KIRC subgroups.** (**A**–**L**) The box plots illustrate the IC50 values of 12 commonly used chemotherapy drugs among three subgroups. These 12 chemotherapy drugs include: ABT737, axitinib, AZD8055, cisplatin, dasatinib, gefitinib, lapatinib, nilotinib, savolitinib, sorafenib, vorinostat, ruxolitinib. The box plot highlights statistically significant differences in drug sensitivity between different KIRC subgroups. Abbreviations: KIRC: kidney renal clear cell carcinoma.

Given the prominence of molecular targeted therapy in KIRC treatment, the utilization of meticulously precise pharmaceutical agents tailored to the unique attributes of therapeutic targets holds considerable promise. Leveraging the “oncoPredict” toolkit, we anticipated the IC50 concentration values for individual KIRC patients. This approach enabled the assessment of LDCD-RGs expression within three distinct KIRC subgroups in response to a panel of twelve drugs, thereby facilitating a personalized treatment strategy for KIRC patients. Among the twelve drugs were compounds commonly employed in renal cancer targeted therapy as well as agents intended for broader cancer treatment applications. The discernible variations in drug sensitivity across divergent KIRC subgroups are as follows: ABT737 (Bcl-2 protein inhibitor): C3 > C2 or C1; axitinib: C3 > C2 or C1; AZD8055 (mTOR inhibitor): C3 > C2 > C1; cisplatin: C3 > C2 or C1; dasatinib: C3 > C2 > C1; gefitinib: C3 > C2 or C1; lapatinib: C3 > C1 or C2; nilotinib: C3 > C1 or C2; savolitinib: C1 > C2 > C3; sorafenib: C3 > C1 or C2; vorinostat: C3 > C1 or C2; ruxolitinib: C1 > C2 > C3 (Figure 5A–5L). The outcomes elucidate the potential of LDCD-RGs as valuable guides in the development of targeted interventions against KIRC.

### Correlations between the LDCD-RGs score and tumor immune microenvironment in the 3 clusters

**Figure 6:**
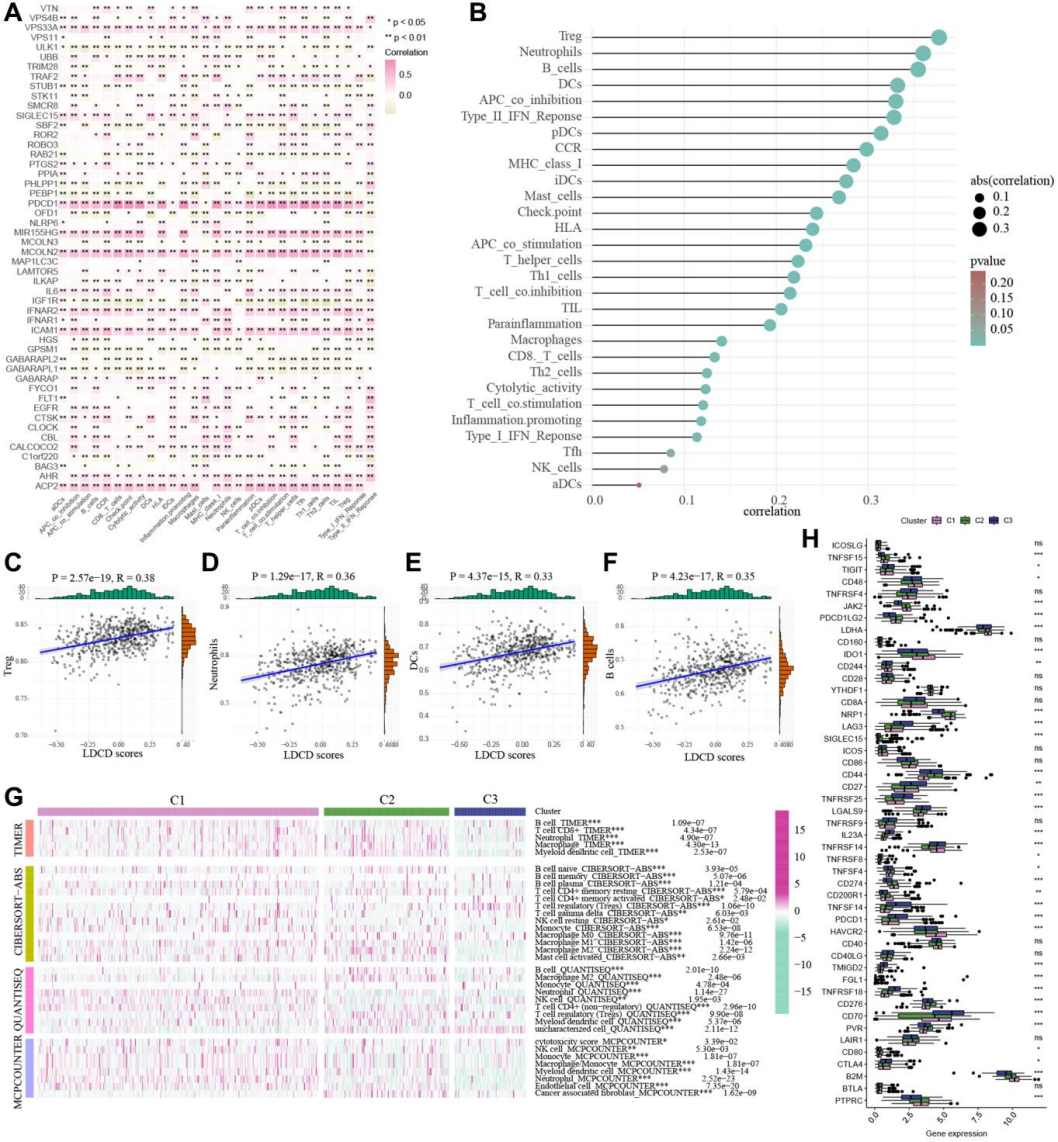
**Correlations between the LDCD-RGs score and tumor immune microenvironment in the 3 clusters.** (**A**) The heat mapping demonstrated a correlation between immune-cell infiltration and LDCD. (**B**) The bubble plot underscored the interconnectedness of immune infiltration-associated cell types with LDCD. (**C**–**F**) The scatter plots illustrating the correlation analysis between LDCD-RGs scores and quantification of four types of immune cell infiltration (Treg cells, neutrophils, B cells, and dendritic cells). All four types of immune cells show a positive correlation with LDCD-RGs scores. (**G**) Heatmap showed the immune cell infiltration levels by TIMER, CIBERSORT, QUANTISEQ, and the MCPCOUNTER algorithm. (**H**) The box plots encompassed the examination of ICGs in 3 clusters. (^*^p < 0.05; ^**^p < 0.01; ^***^p < 0.001; ^****^p < 0.0001). Abbreviations: LDCD-RGs: LDCD-related genes; LDCD: Lysosomal-dependent cell death; ICGs: Immune checkpoint genes.

The tumor microenvironment encompasses an intricate ecosystem comprising of cellular components, extracellular matrix, molecular signals, and vascular structures in proximity to neoplastic cells. Within the context of KIRC, the interplay between the tumor microenvironment and tumor cells significantly influences aspects such as tumor proliferation, metastasis, and resistance to therapeutic interventions [24–26]. A comprehensive comprehension of the mechanisms underpinning the interplay between the tumor microenvironment and tumor cells holds promise for novel insights and strategic approaches in managing KIRC. Notably, an emerging avenue of interest pertains to a novel cell demise pathway known as LDCD, which has garnered substantial attention due to its implications within the tumor microenvironment. Emerging research posits the pivotal role of LDCD in processes such as apoptosis resistance and evasion from immune surveillance in drug-resistant tumor cells. Given these dynamics, a thorough exploration of the relationship between LDCD and the tumor microenvironment stands to be of profound significance in devising innovative anti-tumor therapeutic modalities [27–30]. Through the application of heat mapping (Figure 6A), we demonstrated a correlation between immune-cell infiltration and LDCD. Our investigation unveiled a robust association between LDCD-RGs and immune cell infiltration in individuals with KIRC. Furthermore, visual representations in the form of bubble plots underscored the interconnectedness of immune infiltration-associated cell types with LDCD (Figure 6B). Importantly, our analysis divulged a prevalent positive correlation between the extent of immune cell infiltration and LDCD-RGs. To provide exemplification, we selected the four immune cell types exhibiting the strongest correlations: Treg cells, neutrophils, B cells, and dendritic cells (Figure 6C–6F). Collectively, these findings suggest that individuals with elevated LDCD-RGs scores manifest heightened immune cell infiltration, ultimately translating to a more favorable prognosis within the realm of KIRC. Furthermore, we sought to delve into potential discrepancies in the degree of immune cell infiltration among distinct subgroups, denoted as C1, C2, and C3. Employing computational tools such as TIMER, CIBERSORT, QUANTISEQ, and MCPCOUNTER algorithm, we meticulously evaluated immune cell infiltration levels (Figure 6G). The resulting heat map offered insights into differential cellular immune responses and components across the three subgroups. Notably, the highest infiltration of immune cells was discerned within cluster C1, indicative of a positive correlation between the extent of immune cell infiltration and LDCD-RGs. Considering the pivotal role of immune checkpoints in enabling tumor cells to evade immune surveillance and consequently foster tumor progression, our investigation also encompassed the examination of immune checkpoint genes (ICGs) within varying KIRC subgroups (Figure 6H). Synthesizing the observations, it is plausible to infer that LDCD-RGs wield a significant regulatory influence over the immune milieu in KIRC, thereby holding potential to enhance prognostic outcomes for affected individuals.

### Building and validation of a novel LDCD-RGs related prognostic model for predicting the prognosis of patients with KIRC

**Figure 7:**
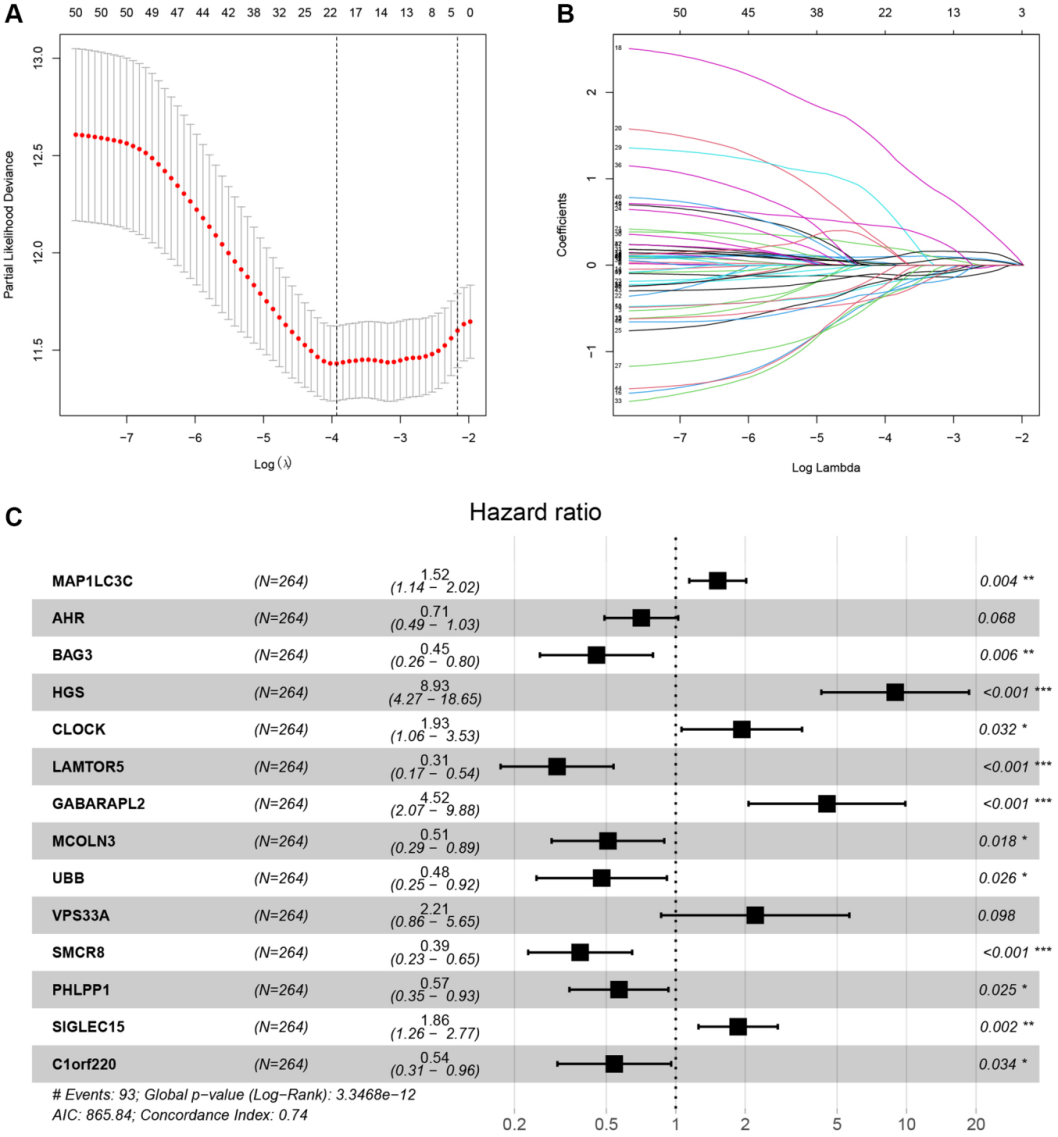
**LASSO-Cox analysis was used for identifying the hub LDCD-RGs.** (**A**) LASSO plot of LDCD-RGs mRNA in KIRC. (**B**) Cross-validation of the constructed model. (**C**) Multivariable Cox regression analysis of LDCD-RGs. Identified 14 target genes for model construction, including: MAP1LC3C, AHR, BAG3, HGS, CLOCK, LAMTOR5, GABARAPL2, MCOLN3, UBB, VPS33A, SMCR8, PHLPP1, SIGLEC15, and C1orf220. Abbreviations: LASSO: Least absolute shrinkage and selection operator; LDCD-RGs: LDCD-related genes; KIRC: kidney renal clear cell carcinoma.

**Figure 8:**
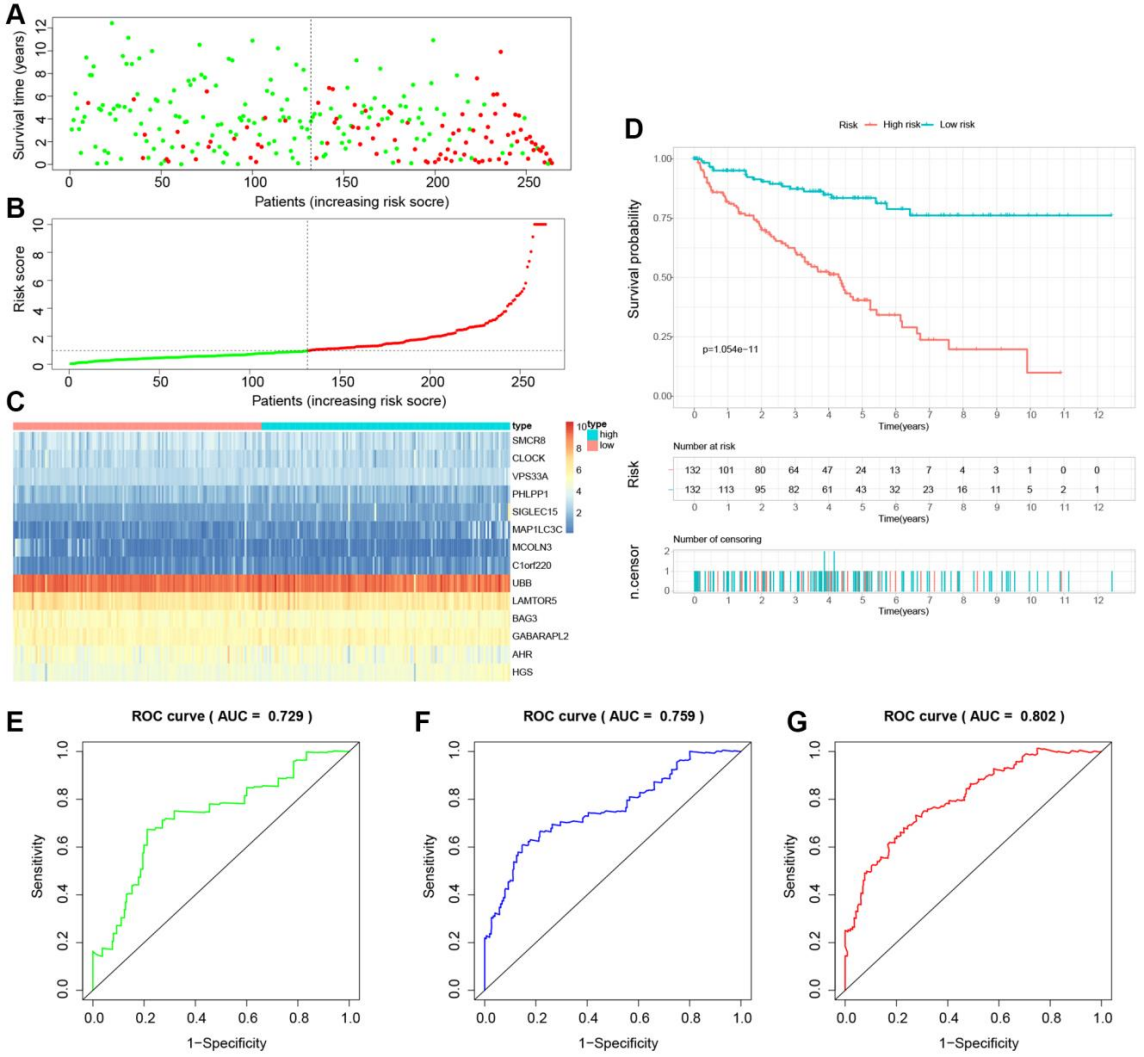
**Building a novel LDCD-RGs related prognostic model for predicting the prognosis of patients with KIRC in the train cohort.** (**A**) KIRC patients were classified according to the median risk score. (**B**) Survival status and risk score distribution of KIRC patients. (**C**) The heatmap displays the expression levels of 14 hub LDCD-RGs between high and low-risk score subgroups. (**D**) Comparison of prognosis in high and low-risk groups of KIRC using Kaplan-Meier survival curve analysis. (**E**–**G**) The AUC values for the ROC curves corresponding to the 1-year, 3-year, and 5-year survival rates were computed at 0.729, 0.759, and 0.802, respectively. Abbreviations: LDCD-RGs: LDCD-related genes; KIRC: kidney renal clear cell carcinoma; AUC: Area Under the Curve.

The study involved a LASSO-Cox regression analysis encompassing 50 LDCD-RGs associated with the prognosis of KIRC. This investigation aimed to discern potential candidate molecules for the establishment of a robust KIRC prognostic framework (Figure 7A, 7B). Subsequently, a comprehensive regression analysis employing the multivariate Cox proportional hazards calculation approach was executed. This step led to the identification of 14 pivotal LDCD-RGs, pivotal for the construction of prognostic models. These genes include MAP1LC3C, AHR, BAG3, HGS, CLOCK, LAMTOR5, GABARAPL2, MCOLN3, UBB, VPS33A, SMCR8, PHLPP1, SIGLEC15, and C1orf220 (Figure 7C). To enhance the precision and applicability of the KIRC prognostic model, a two-fold strategy was adopted for patient selection. Firstly, 50% of the TCGA-KIRC patient cohort was randomly designated as the training set. Concurrently, the remaining 50% of TCGA-IRC patients were used as test1, and all TCGA-KIRC patients were used as test2 (internal validation set). Ultimately, the KIRC patients sourced from the E-MTAB-1980 cohort were employed as an independent external validation set, referred to as test3. In the training cohort, patients were classified into high and low-risk categories based on their median risk scores (Figure 8A). Furthermore, an analysis of the relationship between risk score distribution and survival status was conducted. This analysis unveiled a markedly elevated KIRC mortality rate in the high-risk group as opposed to the low-risk group (Figure 8B). Subsequent to this, a heatmap was presented to visually represent the expression distribution of the 14 LDCD-RGs derived from the prognostic model within the high and low-risk groups (Figure 8C). Following this, the outcomes of the Kaplan-Meier survival analysis substantiated the observation of reduced survival durations among KIRC patients within the high-risk group. Notably, the risk model formulated in the course of this investigation adeptly discerned between unfavorable and favorable prognostic profiles for KIRC cases (Figure 8D). Recognizing the predictive capacity of this risk model concerning KIRC patient prognosis, an assessment of its performance was conducted using ROC curves. The Area Under the Curve (AUC) values for the ROC curves corresponding to the 1-year, 3-year, and 5-year survival rates were computed at 0.729, 0.759, and 0.802 respectively (Figure 8E–8G). Drawing from the aforementioned analyses, it becomes evident that LDCD-RGs hold a pivotal role in orchestrating the tumor immune microenvironment of KIRC. To this end, a diverse array of bioinformatics algorithms was employed to delve into the correlation between immune cell infiltration within the high and low-risk groups (Figure 12A). In sum, it is discerned that within the tumor microenvironment, instances of KIRC in the high-risk category exhibit a heightened extent of immune cell infiltration, with a notable presence of B cells, as opposed to their low-risk counterparts. Conversely, instances of KIRC within the high-risk grouping manifest a diminished level of immune cell infiltration, including Tregs which are associated with immune suppression, in comparison to the low-risk grouping. These findings collectively furnish a rationale for the superior prognosis observed among the high-risk group in relation to the low-risk cohort, thereby further underscoring the profound influence of LDCD-RGs on the overall outcomes of KIRC patients.” into “The analysis of the tumor immune microenvironment highlights significant differences between high and low-risk groups of KIRC patients, particularly in the infiltration of B cells and Treg cells, showing evident heterogeneity. Variations in the abundance of immune cells may be one of the potential reasons for the diverse prognosis observed in these patients.

**Figure 9:**
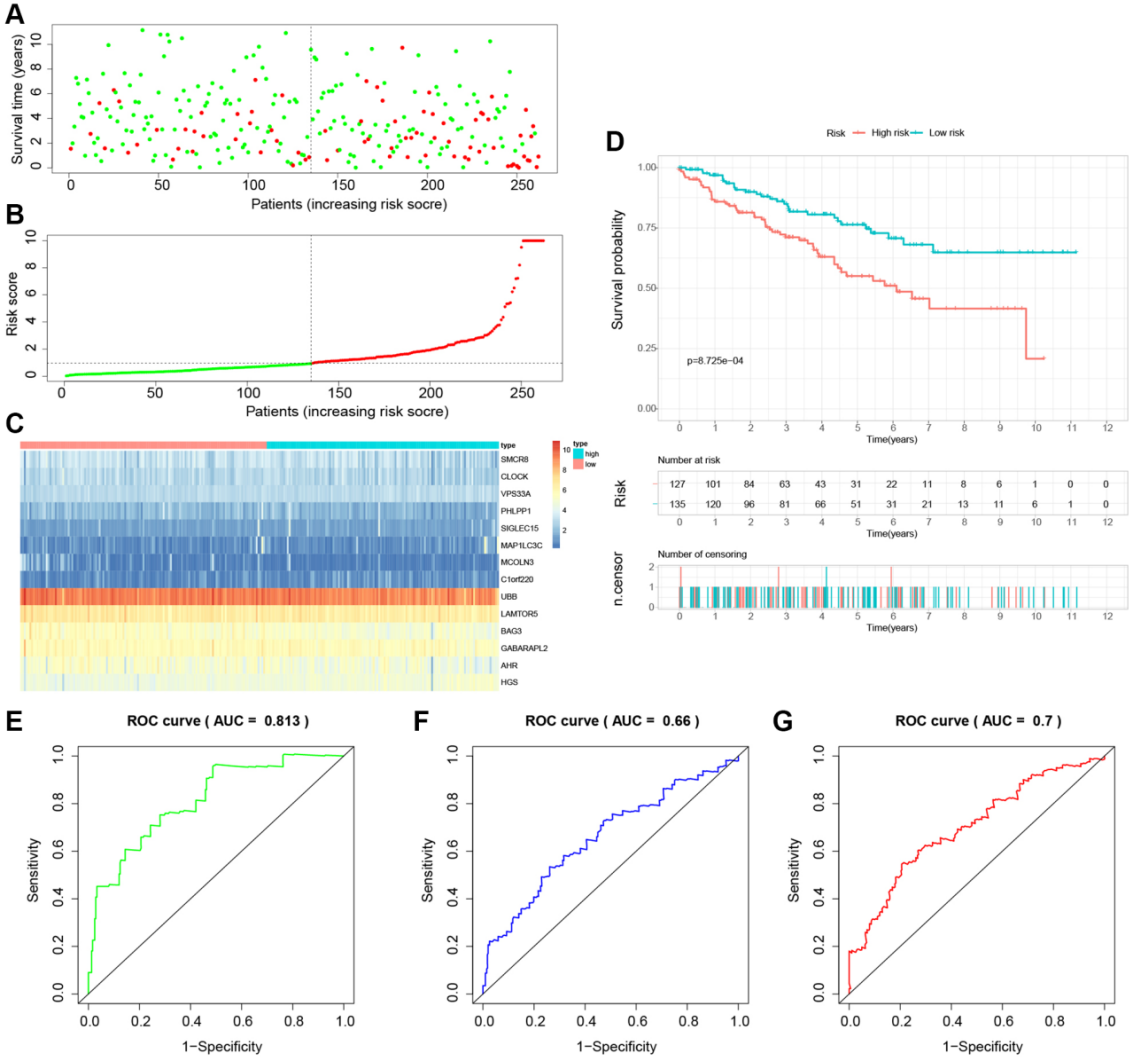
**Internal validation of the novel LDCD-RGs related prognostic model in the test1 cohort.** (**A**) KIRC patients were classified according to the median risk score. (**B**) Survival status and risk score distribution of KIRC patients. (**C**) The heatmap displays the expression levels of 14 hub LDCD-RGs between high and low-risk score subgroups. (**D**) Comparison of prognosis in high and low-risk groups of KIRC using Kaplan-Meier survival curve analysis. (**E**–**G**) The AUC values for the ROC curves corresponding to the 1-year, 3-year, and 5-year survival rates were computed at 0.813, 0.660, and 0.700, respectively. Abbreviations: LDCD-RGs: LDCD-related genes; KIRC: kidney renal clear cell carcinoma; AUC: Area Under the Curve; ROC: Receiver Operating Characteristic.

**Figure 10:**
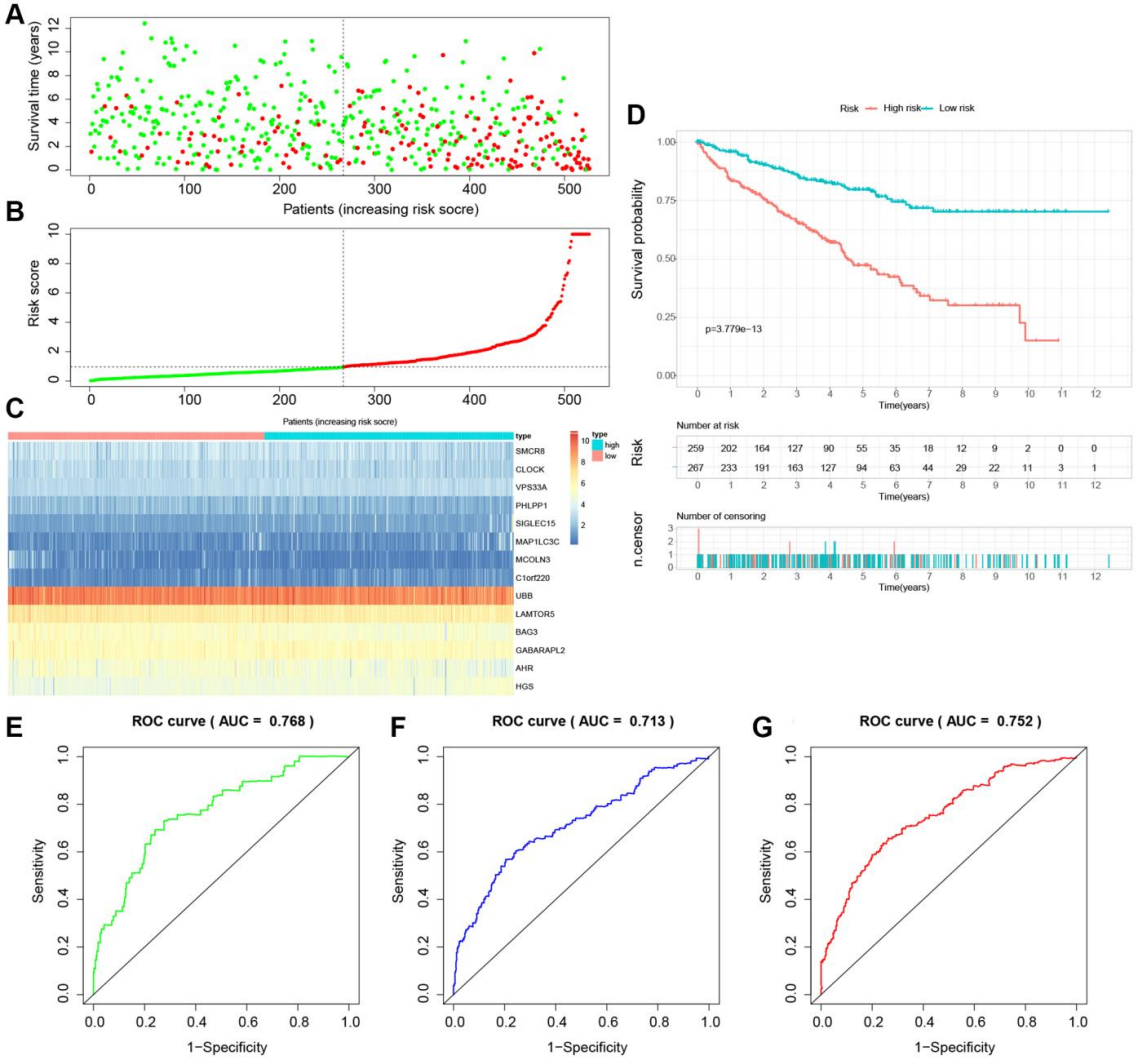
**Internal validation of the novel LDCD-RGs related prognostic model in the test2 cohort.** (**A**) KIRC patients were classified according to the median risk score. (**B**) Survival status and risk score distribution of KIRC patients. (**C**) The heatmap displays the expression levels of 14 hub LDCD-RGs between high and low-risk score subgroups. (**D**) Comparison of prognosis in high and low-risk groups of KIRC using Kaplan-Meier survival curve analysis. (**E**–**G**) The AUC values for the ROC curves corresponding to the 1-year, 3-year, and 5-year survival rates were computed at 0.768, 0.713, and 0.752, respectively. Abbreviations: LDCD-RGs: LDCD-related genes; KIRC: kidney renal clear cell carcinoma; AUC: Area Under the Curve; ROC: Receiver Operating Characteristic.

**Figure 11:**
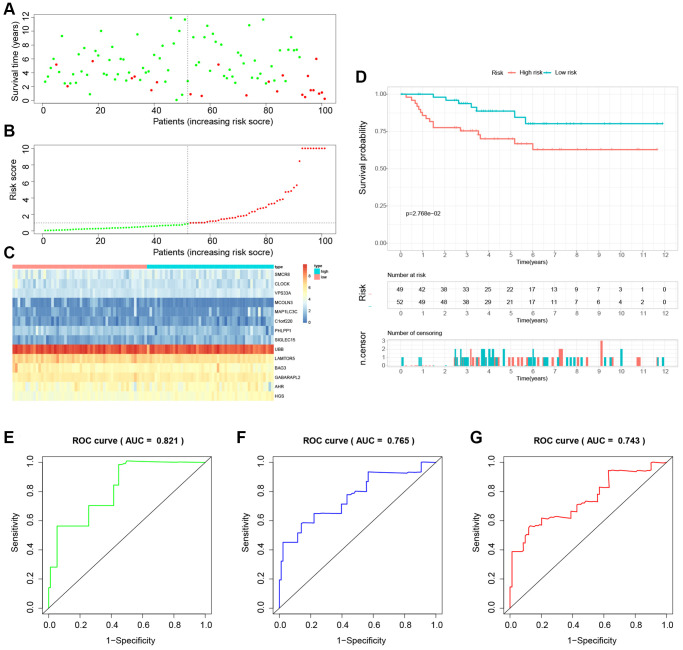
**External validation of the novel LDCD-RGs related prognostic model in the test3 cohort.** (**A**) KIRC patients were classified according to the median risk score. (**B**) Survival status and risk score distribution of KIRC patients. (**C**) The heatmap displays the expression levels of 14 hub LDCD-RGs between high and low-risk score subgroups. (**D**) Comparison of prognosis in high and low-risk groups of KIRC using Kaplan-Meier survival curve analysis. (**E**–**G**) The AUC values for the ROC curves corresponding to the 1-year, 3-year, and 5-year survival rates were computed at 0.821, 0.765, and 0.701, respectively. Abbreviations: LDCD-RGs: LDCD-related genes; KIRC: kidney renal clear cell carcinoma; AUC: Area Under the Curve; ROC: Receiver Operating Characteristic.

**Figure 12:**
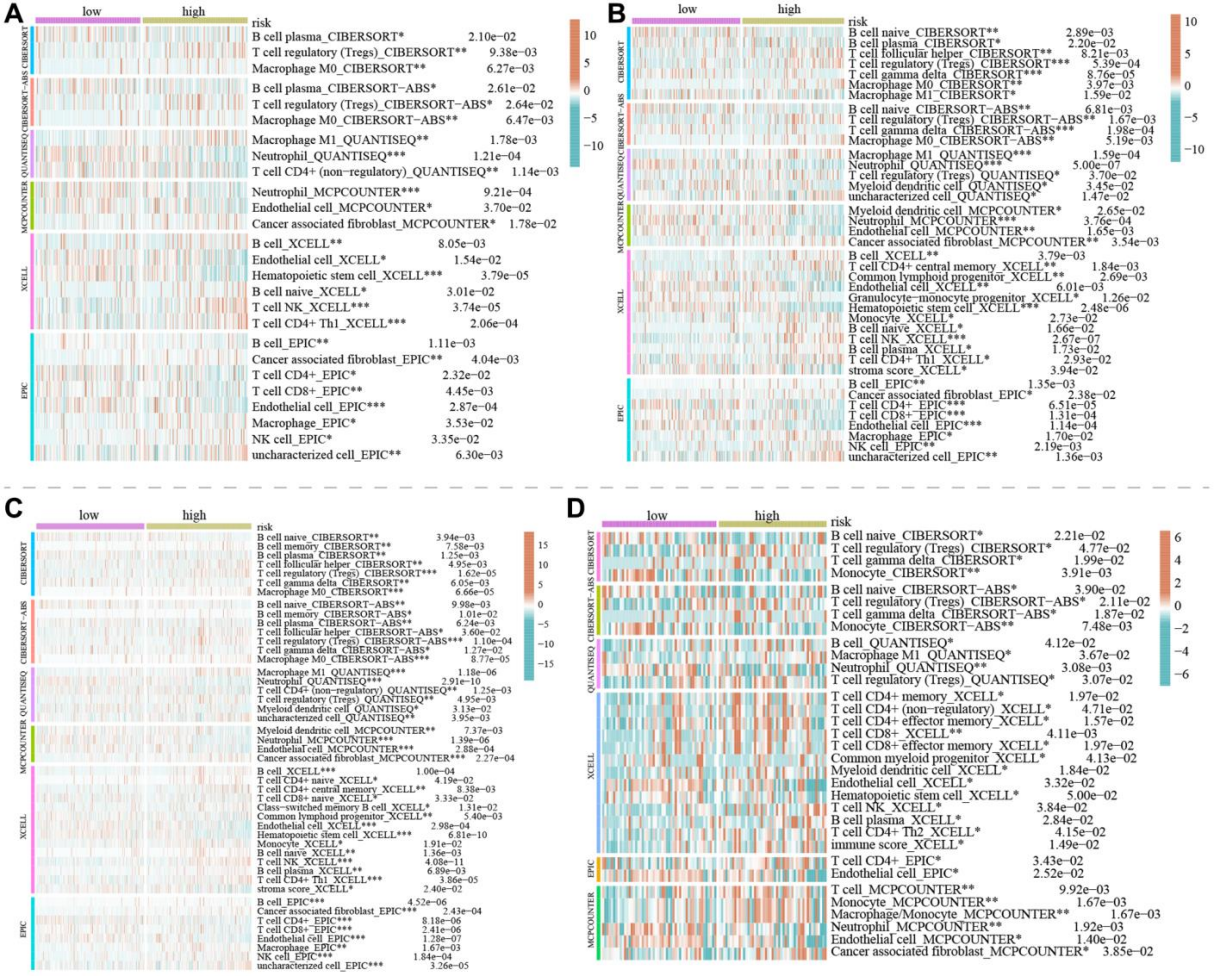
**The correlation between immune cell infiltration between the high and low-risk groups in KIRC.** (**A**–**D**) The degree of immune cell infiltration within the high and low-risk groups by bioinformatics algorithms in the train, test1, test2 and test3 cohorts, respectively. Of note, in both the training and validation sets, the types and extent of immune cell infiltration are similar. (^*^p < 0.05; ^**^p < 0.01; ^***^p < 0.001; ^****^p < 0.0001). Abbreviation: KIRC: kidney renal clear cell carcinoma.

**Figure 13:**
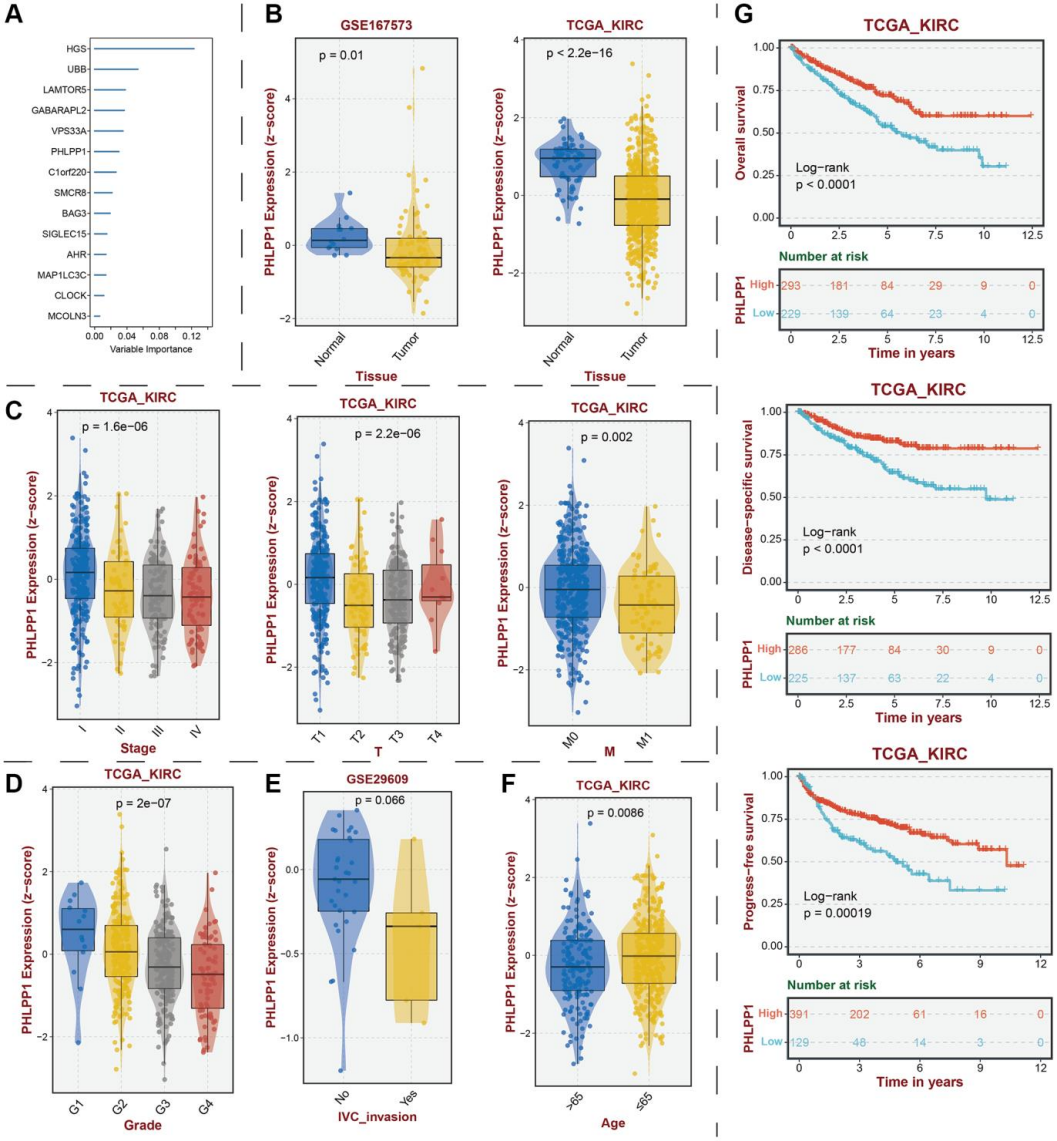
**Identification of key protective protein PHLPP1 in KIRC.** (**A**) Prognostic contribution of model genes predicted by random forest algorithm. (**B**) Transcriptomic expression levels of PHLPP1. (**C**) Association between PHLPP1 expression levels and tumor stage. Association of PHLPP1 expression levels with (**D**) grade, (**E**) IVC invasion, and (**F**) age. (**G**) Association of PHLPP1 expression levels with prognostic indicators for KIRC. Abbreviations: KIRC: kidney renal clear cell carcinoma; IVC: Inferior Vena Cava.

To establish the reliability, stability, and scientific validity of the risk model associated with LDCD-RGs, we applied uniform analytical methodologies to categorize KIRC samples from both internal validation sets (test1, test2) and an external validation set (test3) into distinct low-risk and high-risk groups (Figures 9A, 10A, 11A). Notably, the analysis of both internal and external validation sets revealed that the median risk scores from the training set consistently served as a benchmark. Moreover, the distribution of risk scores and survival statuses across the three validation sets (test1, test2, test3) closely mirrored the outcomes derived from the training set (Figures 9B, 10B, 11B). In both the internal and external validation sets, the expression profiles of the 14 LDCD-RGs within the high-risk and low-risk groups closely paralleled those observed in the training set (Figures 9C, 10C, 11C). Notably, the high-risk groups within all three validation cohorts exhibited unfavorable trends in overall survival (OS) (Figures 9D, 10D, 11D). ROC curve analysis indicated that within the test1 cohort, the AUC values were 0.813, 0.660, and 0.700, respectively (Figure 9E–9G). Within the test2 cohort, the corresponding AUC values were 0.768, 0.713, and 0.752 (Figure 10E–10G). The external validation, represented by the test3 dataset, yielded AUC values of 0.821, 0.765, and 0.743 for the ROC curves (Figure 11E–11G). Collectively, these findings underscore the diagnostic efficacy of the KIRC risk model established in this study. Furthermore, utilizing identical analytical methodologies, we validated immune cell infiltration patterns in test1, test2, and test3, which closely aligned with outcomes observed in the training set (Figure 12B–12D). This correspondence validates the rigor and precision of our analytical approach. Random forest-based survival analysis highlighted the crucial role of PHLPP1 in KIRC (Figure 13A). Although PHLPP1 was not the gene with the highest contribution to prognosis, it ranked among the top genes, underscoring its significant role. Importantly, we found that the genes ranking higher in importance than PHLPP1 were not closely associated with clinical features such as tumor stage and grade. Therefore, our focus was directed towards the PHLPP1 gene. Clinical relevance analysis from the BEST website (Figure 13B–13F) indicated a noticeable downregulation of PHLPP1 gene expression in tumor tissues. Furthermore, as the tumor worsens, there was a further decline in PHLPP1 gene expression, especially during distant metastasis, where this trend became more pronounced. This implied that PHLPP1 was a crucial protective protein in KIRC, aligning with the earlier results of the univariate Cox regression analysis in this study. Additionally, Kaplan-Meier survival analysis validated this viewpoint (Figure 13G). HPA platform revealed that protein level of PHLPP1 in KIRC was obviously lower than that in normal kidney tissues (Supplementary Figure 2).

## DISCUSSION

In the realm of physiology, lysosomes play a pivotal role in maintaining intracellular homeostasis within cells. The integrity of lysosomal membranes emerges as a critical determinant of cellular destiny. Perturbation in lysosomal function underpins various human ailments, including cancer [11, 31, 32]. LDCD signifies a regulated form of cellular demise orchestrated by the discharge of hydrolytic enzymes (cathepsins) or iron from lysosomes, characterized distinctly by the rupture of lysosomal membranes [33, 34]. Research findings have unveiled that impaired lysosomal activity and inhibition of autophagy synergistically contribute to the cytotoxic demise of cervical cancer cells induced by autophagy-linked anticancer peptides, operating through the AMPK/mTOR signaling axis. The manipulation of lysosomal function via inducing permeabilization of lysosomal membranes by chloroquine emerges as a strategy to sensitize refractory non-small cell lung cancer cells to cisplatin [14, 15]. An analogous strategy involving salinomycin orchestrates the sequestration of iron within lysosomes, prompting lysosomal membrane permeabilization and consequent elimination of tumor cells [34]. The pioneering work of Xiwang Yang and collaborators introduced the prospective utility of the epigenetic transcriptional regulator LDCR in governing lysosome-mediated cell demise in lung adenocarcinoma (LUAD). Their investigation delineated the regulatory framework and clinical implications of LDCR, shedding light on targeted intervention of lysosomal processes as a promising avenue in tumor therapeutics [9]. However, the domain of LDCD in the context of tumors remains constrained, leaving the mechanisms underpinning lysosome-mediated survival of cancer cells within tumors inadequately understood. Notably, the role of LDCD in the genesis and progression of KIRC, along with the potential targeted lysosomal strategies for KIRC, remains largely uncharted. There exists an unmet need to explore the pertinent molecular targets and mechanisms. Consequently, a comprehensive exploration involving molecular and immuno-related analyses of KIRC based on genes associated with LDCD holds intrinsic value and potential. This endeavor promises fresh perspectives and a robust theoretical foundation, enriching the landscape of targeted therapeutic approaches for KIRC.

In our study, we have presented, for the first time, the single-cell atlas of LDCD signal in KIRC. Various cell types within the renal microenvironment were identified and quantified. The LDCD cell signals within the tumor tissue were significantly stronger than the cell signals in the adjacent normal tissue. We also emphasized the importance of LDCD-RGs in relation to pan-cancer characteristics across human solid tumors. These attributes encompass CNV, SNV, expression profiles, prognostic implications, and methylation patterns. By means of a comprehensive pan-cancer analysis, we have discerned the pivotal roles assumed by LDCD-RGs across diverse solid tumors. These roles involve either fostering or suppressing tumorigenesis in distinct human malignancies. Given the limited extant research on LDCD-RGs in human tumors, our investigation into this expansive pan-cancer landscape furnishes invaluable guidance for subsequent fundamental scientific inquiry into LDCD-RGs. For instance, within the context of KIRC, the preponderance of LDCD-RGs exhibits altered expression levels. Notably, the specific LDCD-RG referred to as ICAM1 experiences upregulation in KIRC. Noteworthy study has documented an interaction between RARRES1 and ICAM1 that modulates macrophage-mediated inhibition of KIRC progression [35]. This observation thus corroborates the precision of our analysis. Additionally, it is germane to underscore that nearly all LDCD-RGs identified in human tumors manifest a state of low methylation, a phenomenon particularly conspicuous in the context of KIRC. The regulatory capacity of methylation levels on LDCD-RGs in the genesis and advancement of tumors, especially within the domain of KIRC, beckons further exploration.

Subsequently, by leveraging Cox regression analysis, we have pinpointed 50 LDCD-RGs linked to the prognosis of KIRC. Through the application of clustering analysis, we have effectively classified KIRC samples into three distinct subgroups: cluster1, cluster2, and cluster3. Among these subgroups, statistically noteworthy discrepancies in the expression levels of LDCD-RGs have come to light. Our findings divulge that heightening expression of LDCD-RGs in KIRC contributes to the elongation of the survival span for patients afflicted by KIRC. This approach to stratification assumes paramount importance and relevance in the assessment of prognoses and the provision of clinical guidance for KIRC patients. In consonance with the outcomes stemming from the pan-cancer analysis, we have further authenticated the pivotal role assumed by LDCD-RGs in the instigation and progression of KIRC.

It is acknowledged that tumor-associated metabolic and immune pathways play a pivotal role in both the prognosis and development of human tumors. The activation or inhibition of these pathways significantly impacts the clinical outcomes and prognosis of tumor patients [28, 36]. Regulatory mechanisms involving effector molecules in programmed cell death encompass a cascade of signaling reactions, including necrotic apoptosis, pyroptosis, ferroptosis, autophagy, and LDCD. Each of these mechanisms possesses distinct biochemical, morphological, and immunological characteristics [9, 37]. Several regulatory mechanisms of cell death (RCD) have been extensively researched and have demonstrated their indispensability and efficacy in cancer therapy [38]. To gain deeper insights into the underlying reasons for prognostic variations among the three subgroups of KIRC and to elucidate the specific mechanisms through which LDCD-RGs influence the survival rate of KIRC patients, we conducted an analysis of the disparities in classical cancer-related metabolic, immune, and cell death pathways among these 3 subgroups. As expected, notable differences emerged in the aforementioned pathways and cell death modalities across the subgroups. We posit that these disparities potentially underlie the risk-associated role of LDCD in KIRC. Of particular interest, within the scope of this study, C1 characterized by relatively active tumor-related metabolic pathways and comparatively suppressed immune-related pathways, exhibited the most favorable prognosis among KIRC patients. This observation diverges from conventional perspectives. This contrarian outlook could arise from incomplete research into tumor-related metabolic and immune pathways or may be linked to factors such as the age of KIRC patients, tumor staging, tumor grading, the tumor immune microenvironment, or other unidentified mechanisms. During the process of LDCD, changes in lysosomal membrane permeability occur, establishing an association between LDCD and various cell death pathways. We visually depict the interconnectedness of LDCD with other cell death modalities using a heatmap, thereby confirming the proposition that LDCD contributes to the intricacy of cell death pathways [9]. Furthermore, we observe that more than half of the cell death modalities exhibit a negative positive correlation with LDCD. This correlation could also contribute to the favorable prognosis observed in the C1 subgroup.

In the context of KIRC treatment, the continuous progress and advancement in the field of medicine have led to the integration of targeted therapies and immune checkpoint blockade treatments into our clinical strategies [3, 39–43]. Consequently, an in-depth investigation was conducted into the therapeutic efficacy of classical targeted medications across the three distinct subgroups of KIRC. An assessment was undertaken to discern variations in the expression of immune checkpoint molecules within these 3 subgroups. Analysis of drug prediction outcomes has illuminated that, among the cohort of 12 targeted drugs, there exists a differential sensitivity pattern among KIRC patients belonging to diverse subgroups. This observation underscores the potential for personalized therapeutic regimens tailored not only to the individual LDCD-RGs score but also contingent on the selection of specific targeted agents. To illustrate, the utilization of AZD8055 (an mTOR inhibitor) and sorafenib may confer heightened advantages for patients exhibiting subdued LDCD-RGs activity. Conversely, patients with elevated blood LDCD-RGs activity might find ruxolitinib and savolitinib more propitious. Evidently, the conceptualization and development of innovative targeted pharmaceuticals anchored in the LDCD mechanism hold substantial promise within the clinical realm.

The TME refers to the intricate cellular and molecular network present within tumor tissue. This network encompasses a variety of components, including tumor cells, immune cells, blood vessels, and stromal cells. The significance of this microenvironment lies in its pivotal role in regulating tumor initiation, progression, and responses to treatment. Depending on the interactions and equilibrium among its diverse constituents [24, 44], the tumor immune microenvironment can have either positive or negative effects on tumors. Furthermore, a close interrelationship exists between TME and immune checkpoints, with these two entities mutually influencing and co-regulating the immune response to tumors. Upon establishing a correlation between immune cells and LDCD-RGs, it became evident that the extent of immune cell infiltration positively correlates with LDCD-RGs scores. By applying various bioinformatics techniques, we conducted a comprehensive analysis of immune cell infiltration across distinct KIRC 3 subgroups. This analysis revealed heightened immune cell infiltration associated with enhanced immune cells within the C1 subgroup, such as the B cells and T cells, in contrast to the C2 and C3 subgroups. Simultaneously, in the C3 subgroup of KIRC, the expression levels of well-known immune checkpoints, such as CTLA-4, PDCD1, and LAG3, were significantly elevated when compared to the C1 and C2 subgroups. Studies have consistently indicated that increased expression of CTLA-4, PDCD1, and LAG3 portends an unfavorable prognosis for KIRC [45–48]. This underscores the importance of elevated expression of LDCD-RGs, signifying heightened immune cell infiltration. Ultimately, this elevation contributes to an augmented immune response and improved patient prognosis.

Based on the aforementioned conclusions and findings, it can be deduced that LDCD-RGs play a significant role in the progression of KIRC. Nevertheless, owing to the molecular heterogeneity inherent in LDCD-RGs, the precise classification of each individual LDCD-RG fails to reliably prognosticate the outcome for each patient. As a result, leveraging the information from LDCD-RGs, we have devised a risk prediction model with the capacity to accurately anticipate the survival duration of KIRC patients. Our methodology encompassed the utilization of LASSO-Cox regression analysis to identify pivotal molecular constituents for the construction of the model, resulting in the incorporation of 14 LDCD-RGs (namely, MAP1LC3C, AHR, BAG3, HGS, CLOCK, LAMTOR5, GABARAPL2, MCOLN3, UBB, VPS33A, SMCR8, PHLPP1, SIGLEC15, and C1orf220) within the prognostic framework. Validation of our prognostic predictions was substantiated by ROC curves within the training dataset, affirming the precision of our projections for the 1-year, 3-year, and 5-year survival probabilities among KIRC patients. This attests to the clinical predictive efficacy inherent in our model. Further validation was conducted through both internal and external validation sets. Our model’s effectiveness enabled the stratification of KIRC patients into distinct high- and low-risk categories. Kaplan-Meier analysis consistently underscored that the overall survival rates of KIRC patients in the high-risk group were markedly lower than those observed in the low-risk cohort, a pattern that was consistently mirrored in both the training and validation sets. Additionally, an in-depth exploration of immune cell infiltration discrepancies between the high- and low-risk categories was performed, elucidating the variations in immune cell profiles and offering insights into potential rationales for the less favorable prognosis encountered in the high-risk stratum. Across the training set, internal validation set, and external validation set, we validated the scientific validity, rigor, and reliability of our novel LDCD-RGS-related risk models. This model can independently predict the prognosis trajectory of KIRC patients, thereby providing valuable guidance for clinical decision-making. It also provides a theoretical basis for selecting the appropriate treatment strategy according to the situation of each patient.

As a constituent member of the prognostic model, we discovered that PHLPP1 had significant research value in KIRC. The transcription and protein levels of PHLPP1 showed a pronounced decrease in tumor tissues, and this expression level further decreased with age and the malignant progression of the tumor. Especially when patients underwent distant metastasis, the changes in PHLPP1 expression were highly apparent. At that time, there was no research that unveiled the specific role of PHLPP1 in KIRC, but based on existing results, we speculated that it might have exerted anti-tumor effects by inhibiting tumor proliferation, migration, and invasion. Additionally, the regulatory capability of PHLPP1 on the immune microenvironment in KIRC remained unknown. Future studies on this gene were considered to hold substantial value and significance.

Undoubtedly, this study is not without limitations and deficiencies. To begin with, the primary data utilized in this study were extracted from publicly available databases, lacking the inclusion of self-generated data stemming from our dedicated research center. In order to authenticate the clinical applicability of our discoveries, it becomes imperative to conduct indispensable molecular biology experiments alongside clinically pertinent prospective investigations. Furthermore, the exact mechanisms by which the 14 LDCD-RGs included in the prognostic model exert their regulatory influence on the onset and advancement of KIRC remain, as of yet, undisclosed. Lastly, a more thorough exploration is warranted to fathom the precise modalities through which LDCD-RGs modulate various facets such as tumor metabolism, immune response, the tumor microenvironment, and cellular apoptosis pathways. Despite these acknowledged limitations, it is crucial to recognize the robust points and clinical significance embedded in our research outcomes. Our study, in a pioneering manner, sheds light on the central role played by LDCD-RGs in instigating and advancing human tumors, with a specific focus on KIRC. This endeavor offers an all-encompassing clarification of the molecular attributes associated with LDCD-RGs within the domain of KIRC, marking a significant first. The implications of this study’s findings continue to serve as both a compass and a theoretical foundation for fundamental investigations and clinical interventions revolving around LDCD-RGs in the context of KIRC.

## CONCLUSION

In conclusion, this study provides a systematic elucidation of the central role and prognostic significance of LDCD-RGs in both the onset and progression of KIRC. Concurrently, it illustrates the pathways and mechanisms by which LDCD-RGs could exert influence on the clinical outcomes of KIRC. The LDCD-RGs pinpointed through these research findings hold the potential to serve as valuable therapeutic targets in the domain of KIRC investigation. Of paramount importance, this research introduces an innovative risk model for predicting KIRC prognosis and provides guidance for clinical interventions within the sphere of KIRC treatment.

## Supplementary Materials

Supplementary Figures

Supplementary Tables 1 and 2
