# Supplementary material for: Molecular characteristics and prognostic significances of lysosomal-dependent cell death in kidney renal clear cell carcinoma
Source: Aging (Albany NY). 2024 Mar 7;16(5):4862–88. doi: 10.18632/aging.205639 (PMC10968703; doi:10.18632/aging.205639)
Supplement: Supplementary Figures [file aging-16-205639-s001.pdf]

SUPPLEMENTARY FIGURES

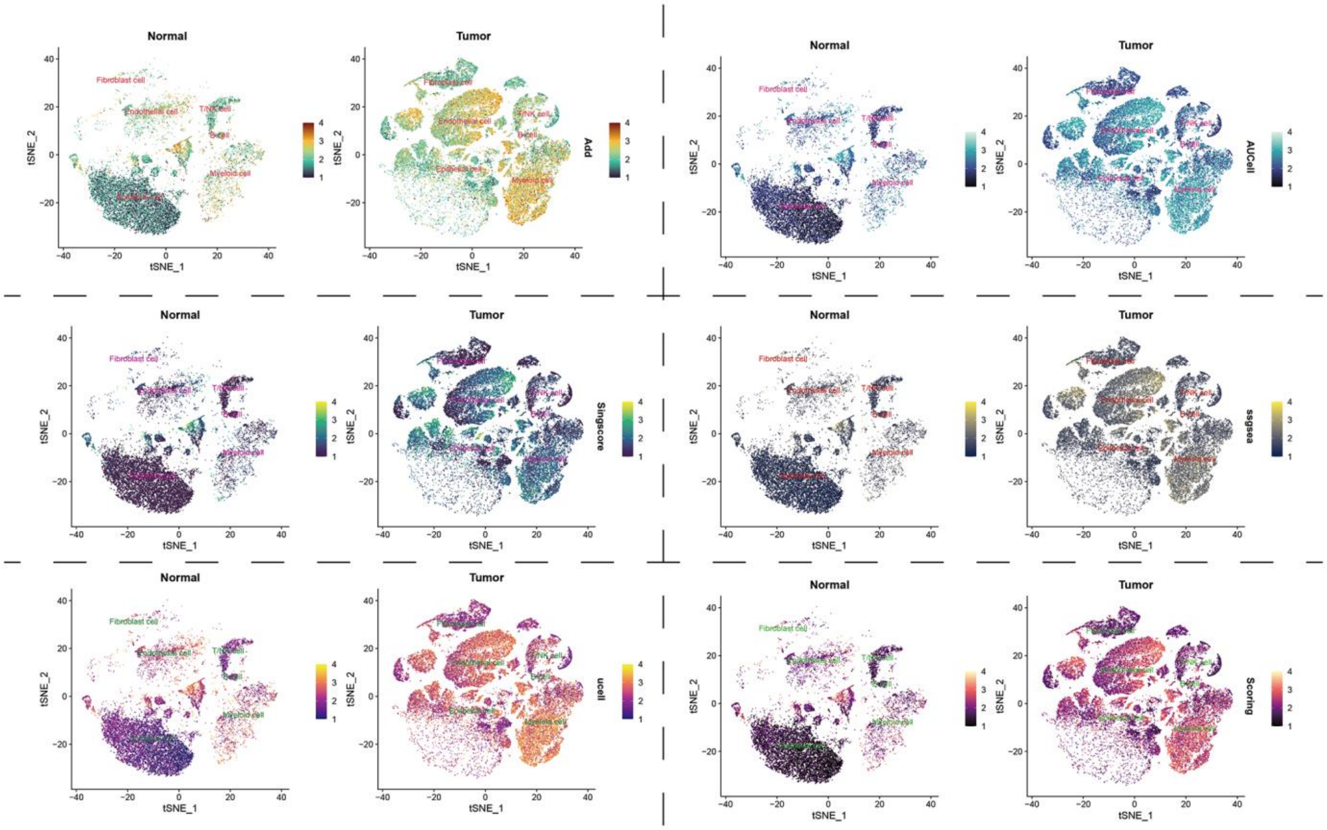

Supplementary Figure 1. LDCD signal characteristics of cancer tissue and normal tissue at single-cell resolution (predicted by Add, AUCell, UCell, ssgsea, singscore, and scoring algorithms). Abbreviation: LDCD: Lysosomal-dependent cell death.

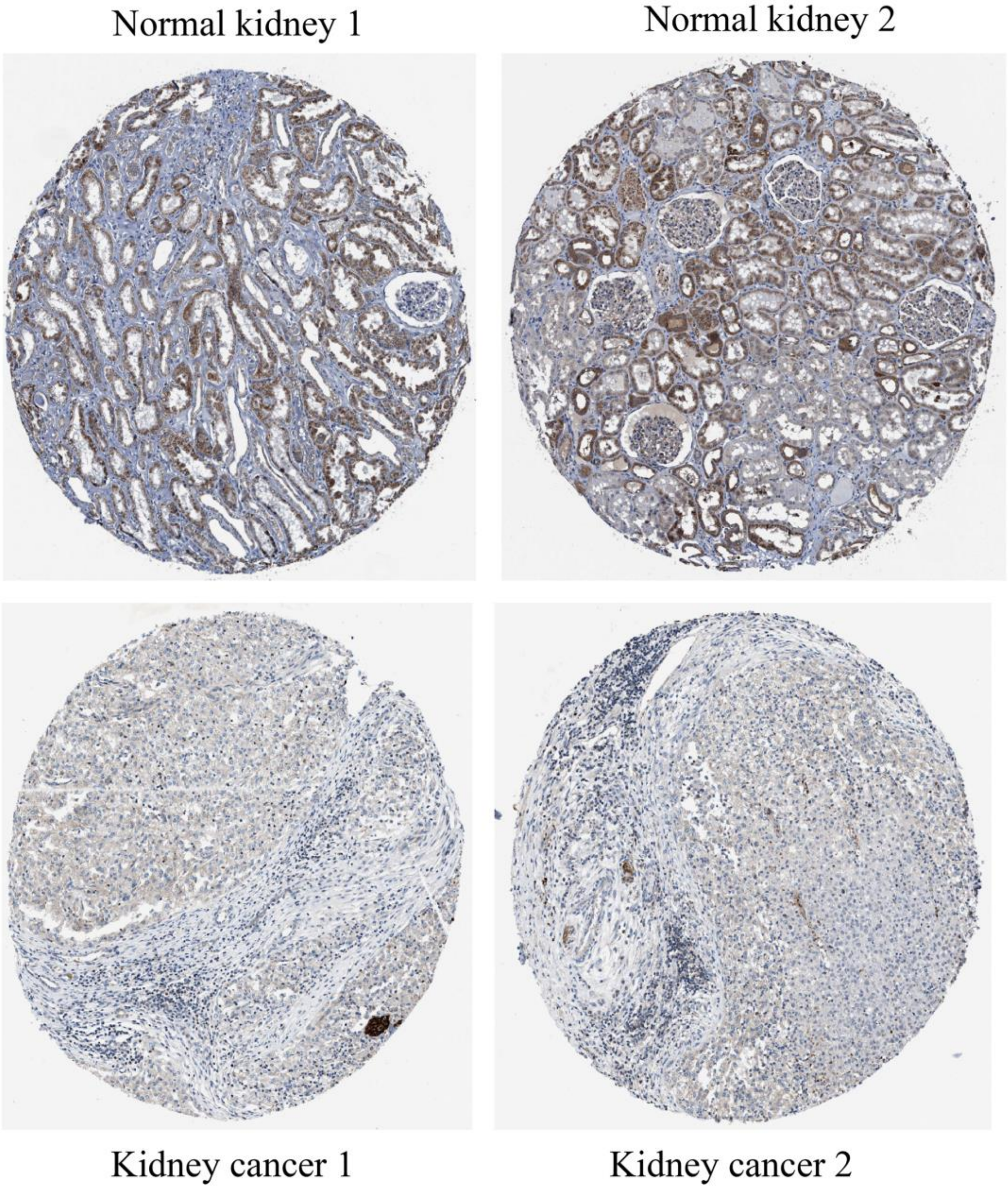

Supplementary Figure 2. Immunohistochemical results of PHLPP1.
